# Carbon-dot-loaded Co_x_Ni_1−x_Fe_2_O_4_; x = 0.9/SiO_2_/TiO_2_ nanocomposite with enhanced photocatalytic and antimicrobial potential: An engineered nanocomposite for wastewater treatment

**DOI:** 10.1038/s41598-020-68173-1

**Published:** 2020-07-13

**Authors:** M. Abd Elkodous, Gharieb S. El-Sayyad, Sally M. Youssry, Hanady G. Nada, Mohamed Gobara, Mohamed A. Elsayed, Ahmed M. El-Khawaga, Go Kawamura, Wai Kian Tan, Ahmed I. El-Batal, Atsunori Matsuda

**Affiliations:** 1 Department of Electrical and Electronic Information Engineering, Toyohashi University of Technology, 1-1 Hibarigaoka, Tempaku-cho, Toyohashi, Aichi 441-8580 Japan; 2 Center for Nanotechnology (CNT), School of Engineering and Applied Sciences, Nile University, Sheikh Zayed, 16453 Giza Egypt; 3 Drug Microbiology Lab, Drug Radiation Research Department, National Center for Radiation Research and Technology (NCRRT), Atomic Energy Authority, Cairo, Egypt; 4 Chemical Engineering Department, Military Technical College (MTC), Egyptian Armed Forces, Cairo, Egypt; 5 Institute of Liberal Arts and Sciences, Toyohashi University of Technology, 1-1 Hibarigaoka, Tempaku-cho, Toyohashi, Aichi 441-8580 Japan

**Keywords:** Microbiology, Chemistry, Materials science, Nanoscience and technology

## Abstract

Water scarcity is now a serious global issue resulting from population growth, water decrease, and pollution. Traditional wastewater treatment plants are insufficient and cannot meet the basic standards of water quality at reasonable cost or processing time. In this paper we report the preparation, characterization and multiple applications of an efficient photocatalytic nanocomposite (Co_x_Ni_1−x_Fe_2_O_4_; x = 0.9/SiO_2_/TiO_2_/C-dots) synthesized by a layer-by-layer method. Then, the photocatalytic capabilities of the synthesized nanocomposite were extensively-studied against aqueous solutions of chloramine-T trihydrate. In addition, reaction kinetics, degradation mechanism and various parameters affecting the photocatalytic efficiency (nanocomposite dose, chloramine-T initial concentration, and reaction pH) were analyzed in detail. Further, the antimicrobial activities of the prepared nanocomposite were tested and the effect of UV-activation on the antimicrobial abilities of the prepared nanocomposite was analyzed. Finally, a comparison between the antimicrobial abilities of the current nanocomposite and our previously-reported nanocomposite (Co_x_Ni_1−x_Fe_2_O_4_; x = 0.9/SiO_2_/TiO_2_) had been carried out. Our results revealed that the prepared nanocomposite possessed a high degree of crystallinity, confirmed by XRD, while UV–Vis. recorded an absorption peak at 299 nm. In addition, the prepared nanocomposite possessed BET-surface area of (28.29 ± 0.19 m^2^/g) with narrow pore size distribution. Moreover, it had semi-spherical morphology, high-purity and an average particle size of (19.0 nm). The photocatalytic degradation efficiency was inversely-proportional to chloramine-T initial concentration and directly proportional to the photocatalyst dose. In addition, basic medium (pH 9) was the best suited for chloramine-T degradation. Moreover, UV-irradiation improved the antimicrobial abilities of the prepared nanocomposite against *E. coli*, *B. cereus*, and *C. tropicalis* after 60 min. The observed antimicrobial abilities (high ZOI, low MIC and more efficient antibiofilm capabilities) were unique compared to our previously-reported nanocomposite. Our work offers significant insights into more efficient water treatment and fosters the ongoing efforts looking at how pollutants degrade the water supply and the disinfection of water-borne pathogenic microorganisms.

## Introduction

Population growth, the continued demand of water and water shortages from one year to another are pushing the world towards a water shortage problem^1–3^. In addition, most of the available potable water on earth is contaminated with many kinds of pollutants such as organic, inorganic materials, heavy metals and pathogenic microorganisms, causing serious diseases^4–7^. Among these pollutants, chloramine-T is gaining a lot of attention due to its adverse effects on public health, and is seriously threatening the aquatic environment^8,9^. Chloramine-T is an organic compound with a chloro-substituent in the place of an amino hydrogen^10^. It is currently-used in many applications such as antifouling biocides, disinfectant, various food products, and different cosmetics^11–13^. However, several toxicological experiments have shown that chloramine-T is harmful to the skin and eyes of humans^14^. In addition, it causes irritation of the upper respiratory tract^15^ and in severe cases nausea, vomiting and diarrhea^16^. Thus, reduction and degradation of chloramine-T into relatively-safe intermediates is important for our environment. Currently, there are many water treatment and purification methods^17–19^. However, these traditional methods are not effective in terms of water quality, time of processing and overall cost of operation. Nanotechnology, through nanomaterials, offers not only more-effective ways of water purification, but also cost-effective and easily-manipulated techniques. Nanomaterials possess outstanding chemical, physical and biological properties compared to their bulk materials, due to the large number of surface atoms compared to the inner mass^20–23^. Recently, there are many reports highlighting various promising nano materials and composites for heavy metal adsorption and dye removal applications^24–28^. With respect to them, titanium dioxide nanoparticles (TiO_2_ NPs) have been extensively- studied over the past decades (since water splitting by Fujishima and Honda in 1972) due to their cost-effectiveness, high degree of thermal and chemical stabilities, high-oxidizing ability, and their good electronic and optical characteristics^29^. TiO_2_ NPs are very promising in many environmental applications like water treatment. However, the technological application of TiO_2_ NPs is limited by two main drawbacks^30^. Firstly, they possess a wide energy bandgap (2.96, 3.02 and 3.2 eV) for the brookite, rutile and anatase phases, respectively^31^. Thus, TiO_2_ NPs are activated only by ultraviolet (UV) light, representing a tiny portion of the total solar spectrum (about 3%)^32^. Secondly, TiO_2_ NPs have short electron–hole recombination time (about 10^–9^ s), while photocatalytic reactions need times at the order of (10^–3^ s), that further deteriorates the photocatalytic efficiency of TiO_2_ NPs^33,34^. Various tailoring methods have been reported for increasing the quantum efficiency of TiO_2_ NPs such as the formation of mixed TiO_2_ phases which reduces the band gap energy^35^, doping with ions of non-metals like carbon^36^ and sulfur^37^ and loading with noble metals like silver (Ag)^38^, platinum (Pt) and gold (Au)^39^ which elongate electron–hole recombination time. Currently, loading TiO_2_ NPs with cheap carbon nanoparticles is gaining significant considerations, such as conjugation with activated carbon which improved the adsorption potential of TiO_2_ NPs^40^. Additionally, loading with graphene and carbon nanotubes has shown to provide good electron mobility and reduced the recombination time of the photo-generated charge carriers^41,42^. Recently, carbon dots (C-dots) have received much interest due to their strong photoluminescence, abundance, cost-effectiveness, ease of manipulation, and chemical inertness, and their role as electron reservoirs which elongates the electron–hole recombination time during photocatalytic degradation^43–46^.

In this study, single nanoparticles were incorporated into one nanocomposite to gain more benefits. A recyclable TiO_2_-based and C-dots-loaded nanocomposite (Co_x_Ni_1−x_Fe_2_O_4_; x = 0.9/SiO_2_/TiO_2_/C-dots) was prepared by a layer-by-layer method. A magnetic core (Co_x_Ni_1−x_Fe_2_O_4_; x = 0.9) was formed to make the nanocomposite recyclable by magnetic separation, SiO_2_ layer was used as a protective shell for the TiO_2_ NPs (to protect the TiO_2_ NPs from the formation of pseudo-brookite due to magnetic core contact), anatase TiO_2_ shell was prepared as an efficient photocatalyst with good photocatalytic abilities, and C-dots were loaded onto the TiO_2_ surface to act as an electron reservoir to increase TiO_2_ quantum efficiency. The photocatalytic abilities of the prepared nanocomposite were tested against chloramine-T degradation (an organic compound). In addition, reaction kinetics, photocatalytic mechanism and different parameters affecting the efficiency of photodegradation (nanocomposite dose, chloramine-T initial concentration and reaction pH) had been analyzed. Moreover, the antimicrobial activities of the synthesized nanocomposite had been tested against multi-drug- resistant bacteria and pathogenic fungi. Finally, the effect of UV-activation on the antimicrobial abilities of the prepared nanocomposite had been studied.

## Materials and methods

### Materials

Copper acetate monohydrate (C_4_H_8_CuO_5_), ascorbic acid (C_6_H_8_O_6_), titanium (IV) isopropoxide 97% (C_12_H_28_O_4_Ti), ammonium hydroxide 28% (NH_4_OH), tetraethyl orthosilicate (TEOS) 98% [Si (OC_2_H_5_)_4_], absolute ethanol 99.9% (C_2_H_5_OH), hydroxypropyl cellulose (M.W. = 80,000), nickel chloride (NiCl_2_), cobalt chloride (CoCl_2_), sodium hydroxide pellets (NaOH), Ferric chloride hexahydrate (FeCl_3_·6H_2_O), and chloramine-T trihydrate C_7_H_7_ClNNaO_2_S·3H_2_O were purchased from Sigma Aldrich (Germany). All reagents were of extra-pure grade and were used as received without further purification.

### Method

#### Preparation of the sandwich structure: (Co_x_Ni_1−x_Fe_2_O_4_; x = 0.9/SiO_2_/TiO_2_) nanocomposite

The detailed preparation steps are reported in our previously-published papers^47,48^.

#### Preparation of carbon nanoparticles (C-dots)

C-dots were prepared according to the method reported by Xiaofang Jia et al.^49^. Briefly, (6.8 g) of ascorbic acid were dissolved in (400 ml) deionized water (D.I.W.). (0.8 g) of copper acetate monohydrate was then added into the above solution, and the solution stirred at room temperature for 10 min. The temperature was then raised to 90 °C and kept for 5 h and the remaining solution centrifuged at 9,000 rpm for 20 min to remove large particles. Finally, the supernatant containing the C-dots was dried at 60 °C for 8 h.

#### Preparation of (Co_x_Ni_1−x_Fe_2_O_4_; x = 0.9/SiO_2_/TiO_2_/C-dots) nanocomposite

**Figure 1:**
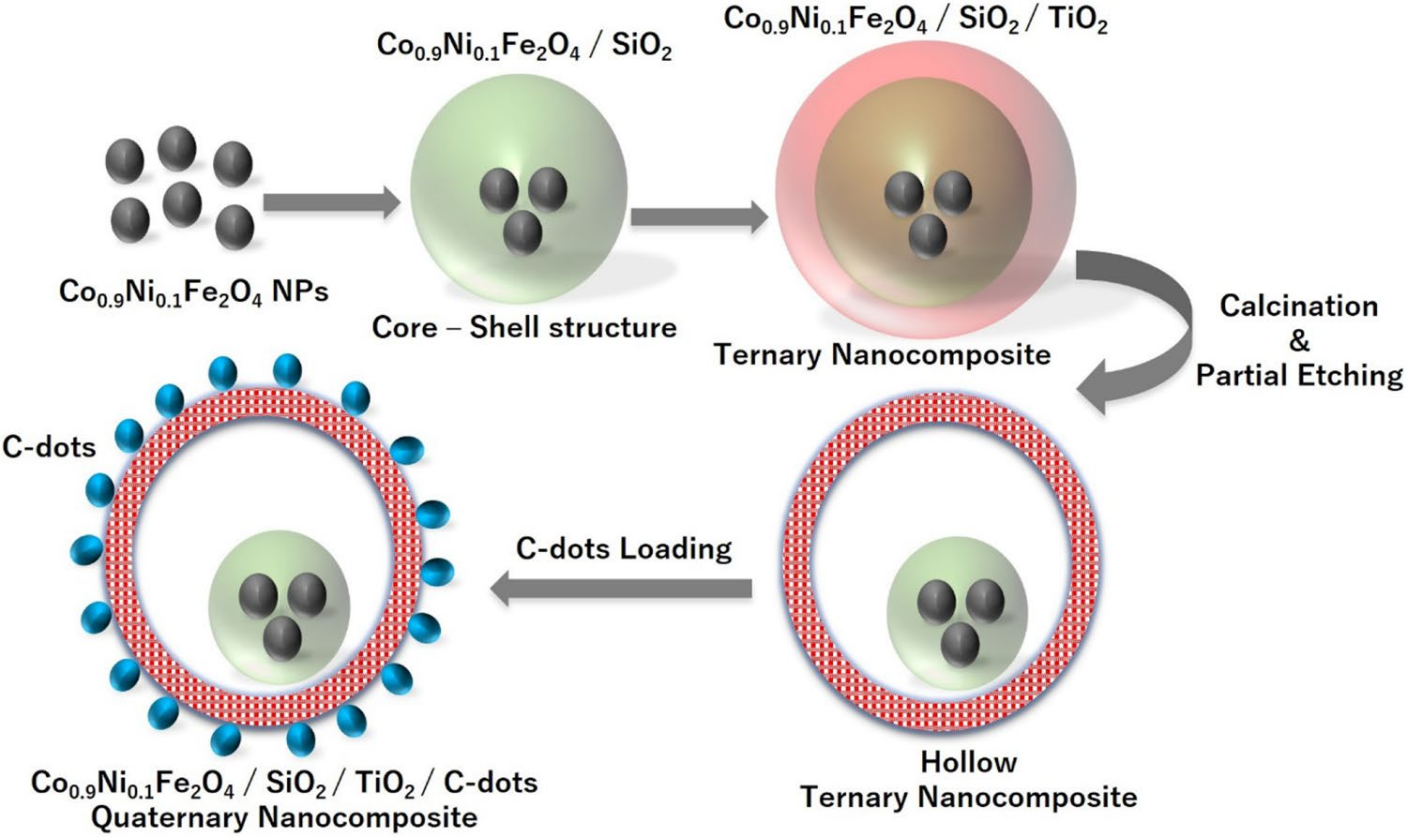
Schematic representation of the mechanism of Co_x_Ni_1−x_Fe_2_O_4_; x = 0.9/SiO_2_/TiO_2_/C-dots nanocomposite formation.

The C-dots-loaded nanocomposite was prepared according to the method reported in our previously-published paper^29^. Briefly, 0.8 g of the nanocomposite from part (Preparation of the sandwich structure) was mixed with 0.1 g of C-dots from part (Preparation of carbon nanoparticles). Then, 50 ml D.I.W was added, and the mixture dispersed using water-bath sonication for 45 min. The hybrid composite was then centrifuged at 8,000 rpm for 15 min. Finally, the collected composite was washed with D.I.W. and dried at 85 °C for 2 h. The preparation steps are schematically-represented in Fig. 1.

#### Characterization of the prepared nanocomposite

Crystallinity and phase were studied using x-ray diffraction (XRD) analysis on an Ultima IVX-ray diffractometer, Rigaku, Japan, applying a voltage of 40 kV, a current of 30 mA and Cu K_α_ radiation (λ = 1.540598 Å). UV–Vis. absorption was calculated via a V-670 spectrophotometer, JASCO, Japan. BET and BJH analyses were used to determine surface area and pore size distribution via Tristar II Micromeritics, Japan. The average particle size was determined by a high-resolution transmission electron microscope (HR-TEM), JEM-2100 F, JEOL Ltd., Japan. The morphology, elemental composition and purity of the particles were analyzed by scanning electron microscope (SEM) supported with an energy-dispersive X-ray (EDX) unit, SU8000 Type II, HITACHI high technologies, Japan. FTIR analysis was carried out by an FT-IR 3600, JASCO Infra-Red spectrometer via the KBr pellet method. It was recorded through a wave-number scale from 4,000 to 400 cm^−1^.

#### Photocatalytic activity of (Co_x_Ni_1−x_Fe_2_O_4_; x = 0.9/SiO_2_/TiO_2_/C-dots) nanocomposite against chloramine-T trihydrate

Photocatalytic experiments were carried out at an ambient temperature of 24 ± 2 °C. A fixed amount of 10 mg of the synthesized nanocomposite was added to a 50 mL aqueous solution of chloramine-T (C_o_ = 10 mg/l) and stirred for 2 h. in the dark. After reaching adsorption–desorption equilibrium, the suspension was illuminated by a low-pressure, 10 W mercury lamp with 90% emittance at 254 nm. The lamp was axially-located and held in a quartz immersion tube. At given irradiation time intervals, 1 ml of the suspension was taken out by a syringe equipped with 2.5 μm pore size filter. The filtered supernatant was centrifuged at (5,000 rpm) for (10 min), to remove particles of the employed photocatalyst. The changes in chloramine-T concentration during photo-decomposition were determined by measuring the absorbance at λ_max_ = 225 nm as a function of irradiation time in the liquid cuvette configuration. D.I.W. was used as the reference on a UV–Vis. spectrophotometer (Agilent Technologies Cary 60 UV–Vis.). The concentration of chloramine-T prior to UV-irradiation was used as the initial value for the measurement of chloramine-T degradation.

#### Antimicrobial activity of C-dots and Co_x_Ni1−xFe_2_O_4_; x = 0.9/SiO_2_/TiO_2_/C-dots nanocomposite

Both C-dots and Co_x_Ni_1−x_Fe_2_O_4_; x = 0.9/SiO_2_/TiO_2_/C-dots nanocomposite were dispersed in DMSO to form two tested concentrations for each sample (10 and 15 µg/ml). Next, their antimicrobial activities were individually-tested using the agar well diffusion method^50^, against different isolates of infection-causing bacteria such as *Staphylococcus aureus* (MRSA), *Escherichia coli*, *Bacillus cereus*, *Pseudomonas aeruginosa*, and *Klebsiella pneumoniae*. Furthermore, the antifungal potentials of both samples were checked against unicellular pathogenic fungi (*Candida tropicalis* and *Candida albicans*). The tested microorganisms were kindly-gifted from the culture collection of Drug Microbiology Lab., Drug Radiation Research Dep., NCRRT, Cairo, Egypt.

It is worth stating that 0.5 McFarland standard of all tested bacterial inoculums was fixed at 3–4 × 10^8^ CFU/ml and 2–5 × 10^8^ CFU/ml for pathogenic yeast. Growth restraint of the examined pathogenic bacteria and yeast was defined by measuring the zone of inhibition (ZOI) after 24 h. of incubation^51^.

In addition, conventional antibiotic discs such as nystatin (NS), with 6 mm-diameter, and a ready-to-use solution of Amoxicillin**/**Clavulanic acid (AMC, 100 µg/ml) were utilized as references to compare the abilities of the developed nanocomposite^52^.

A minimum inhibitory concentration (MIC) was defined using Luria–Bertani (LB) broth with suitable serial dilution^53,54^. A test tube containing the selected microorganism and the nutrient was employed as a positive control, and another tube with just the nutrient was used as a negative control. C-dots and the prepared C-dots loaded-nanocomposite (beginning with a concentration of 50 mg/ml) were examined to determine their MIC values. MIC values were measured after 24 h. of incubation at 37 °C^55,56^. The examined bacterial inoculums were fixed at 3–5 × 10^8^ CFU/ml, while *Candida* species were fixed at 2–5 × 10^7^ CFU/ml. MIC values were determined by ELISA plate reader at absorption wavelength of 600 nm^56,57^.

#### Antibiofilm activity of Co_x_Ni_1−x_Fe_2_O_4_; x = 0.9/SiO_2_/TiO_2_/C-dots nanocomposite

A semi-quantitative investigation of biofilm growth by pathogenic bacteria and yeast and its inhibition by the prepared C-dots loaded nanocomposite were evaluated according to the process described by Christensen et al.^58^. The apparent detection of biofilms created by pathogenic bacteria and yeast throughout the inner walls of test tubes having the tested nanocomposite with a concentration of (15 µg/ml) and tubes without the nanocomposite (control) was carried out.

Additionally, nutrient broth (5 ml) was added to all test tubes after setting 0.5 McFarland standards at 2–3 × 10^7^ CFU/ml (for the tested bacteria). The tubes were then incubated for 24 h. at 37 °C. The content of the control tubes and nanocomposite-included tubes was discarded and tubes were washed and cleaned with phosphate buffer saline (PBS, pH = 7.4). After that, tubes were dried^48,59^. The developed yeast and bacterial biofilms were fixed by 5 ml sodium acetate (3%) for 15 min, and then all tubes were washed with D.I.W. Bacterial and yeast biofilms were dyed with 0.1% crystal violet (CV) for 10 min, then D.I.W. used to eliminate the excess quantity of CV^60^. Additionally, 4 ml of absolute ethanol was employed to dissolve CV. The formed biofilms were identified by the characteristic stained rings around the walls of test tubes^61^. The bacterial and yeast biofilms were investigated using a UV–Vis. spectrophotometer at 570 nm, and the biofilm suppression percentage (%) was determined by utilizing Eq. (1)^48,62^.

1 $${\text{Percentage of bacterial and yeast biofilm inhibition}}\left( \% \right) = \frac{{{\text{O.D. of the control sample}} - {\text{O.D. of the treated sample }}}}{{{\text{O.D. of the control sample}}}} \times 100$$

#### Effect of UV-irradiation on the antimicrobial abilities of the prepared nanocomposite

To distinguish the influence of UV-irradiation on the antimicrobial potential of the synthesized Co_x_Ni_1−x_Fe_2_O_4_; x = 0.9/SiO_2_/TiO_2_/C-dots nanocomposite against microbes, the restraint percentage was defined by measuring the optical density of the viable and dead microbial cells^63^. Three susceptible microbes were selected, including *E. coli* (Gram-negative bacteria), *B. cereus* (Gram-positive bacteria) and *C. tropicalis* (unicellular fungi). For each microorganism, four test tubes were prepared. The first tube was a control, which contained tested microbes and was not UV-irradiated; the second had both tested microbes and the prepared nanocomposite and was not UV-irradiated; the third contained the tested microbes and was UV-irradiated; and the fourth included both tested microbes and the synthesized nanocomposite, and was UV-irradiated.

All four tubes had nutrient broth and a fixed number of microorganisms (0.5 McFarland, CFU/ml). A low-pressure mercury lamp emitting UV (10 W, 90% emittance at 254 nm) was horizontally-positioned and settled on the laminar flow. Examined tubes were subjected to UV-irradiation for 1 h at a distance of about 60.96 cm.

It is worth to mention that the number of bacteria and yeast was determined every 10 min through a UV–Vis. spectrophotometer, at a wavelength of 600 nm for bacteria and 630 nm for *Candida species,* for 1 h and the repression percentage % was estimated by Eq. (1).

#### Reaction mechanism using SEM/EDX analysis of nanocomposite-treated microbial cells

Obtained bacterial and *Candida* cells from the biofilm-forming test were washed with PBS and fixed with 3% glutaraldehyde solution. The preserved bacterial and *Candida* specimens were regularly-washed with PBS and evenly-dehydrated with various concentrations of ethanol (30%, 50%, 70%, 80%, 95%, and 100%) for about 20 min at 28 ± 2 °C^59^. Next, bacterial and *Candida* cells were placed on an aluminum scrap for SEM/EDX analysis^59^. The morphological characteristics of the control (non-treated pathogenic bacteria and yeast) and nanocomposite-treated bacterial and yeast cells were observed using SEM/EDX analysis.

#### Statistical analysis

Statistical interpretation of our results was performed through the ONE-WAY ANOVA analysis (at P < 0.05), using Duncan's multiple series studies, and the least significant difference (LSD) record^64^. The obtained results were also analyzed by SPSS software (version 15).

## Results and discussion

### Characterization of the prepared Co_x_Ni_1−x_Fe_2_O_4_; x = 0.9/SiO2/TiO_2_/C-dots nanocomposite

#### XRD analysis

**Figure 2:**
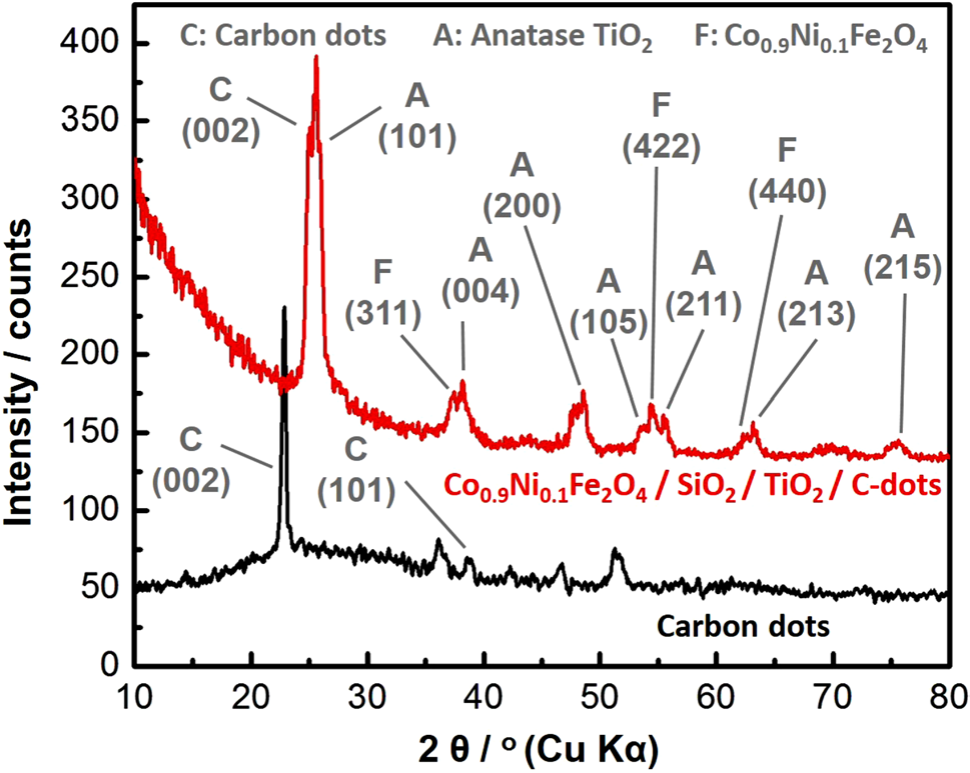
XRD patterns of the prepared C-dots NPs and Co_x_Ni_1−x_Fe_2_O_4_; x = 0.9/SiO_2_/TiO_2_/C-dots nanocomposite.

Crystallinity and phase of the prepared C-dots and the whole nanocomposite were studied using XRD, as depicted in Fig. 2. Several diffraction peaks were recorded, such as the peak at 2θ = 22.9°, plane (002), which corresponds to the C-dots as shown in Fig. 2^65^, while peaks recorded at 2θ = 25.5°, plane (101), 38.1°, plane (004), 48.4°, plane (200), 53.6°, plane (105), 55.4°, plane (211), 63.1°, plane (213), and 75.6°, plane(215) correspond to anatase TiO_2_ NPs (JCPDS 21-1272). In addition, peaks observed at 2θ = 37.2°, plane (311), 54.3°, plane (422), and 62.3°, plane (440) were due to the cobalt and nickel ferrite of Co_x_Ni_1−x_Fe_2_O_4_; x = 0.9 NPs (JCPDS 10–325 and JCPDS 1-1121). It is worth mentioning that an SiO_2_ amorphous halo was suppressed due to the high intensity of C-dots and TiO_2_ peaks, as previously-reported in our paper^48^.

#### UV–Vis. spectroscopic analysis and bandgap calculation

**Figure 3:**
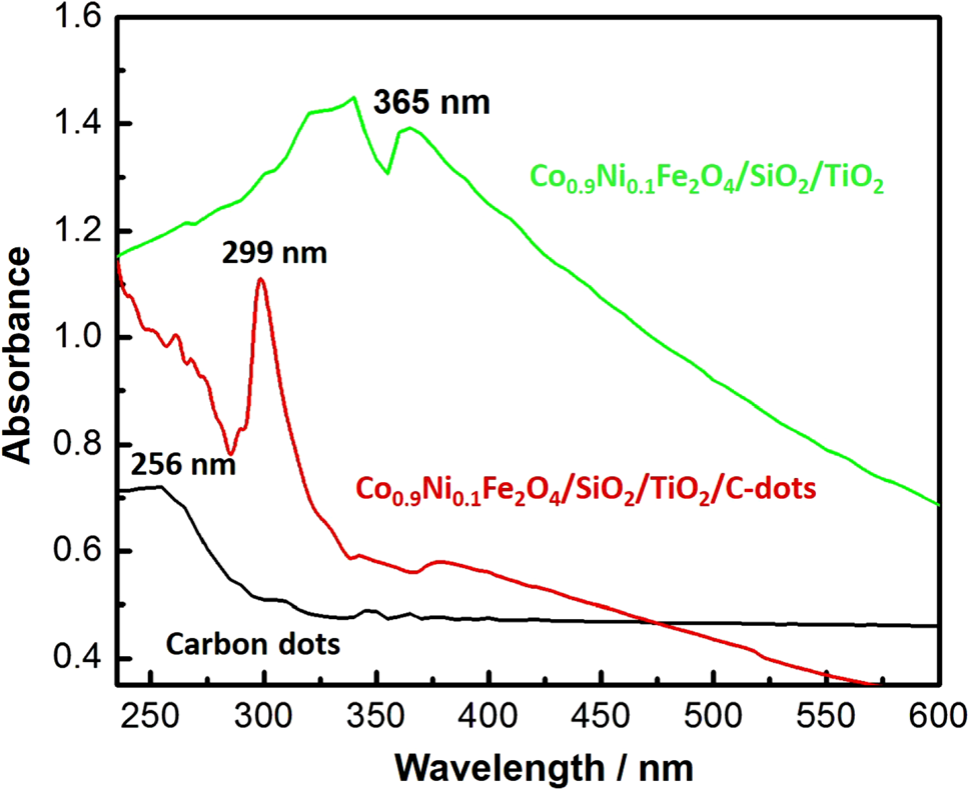
UV–Vis. absorption spectra of the prepared Co_x_Ni_1−x_Fe_2_O_4_; x = 0.9/SiO_2_/TiO_2_/C-dots nanocomposite, bare C-dots and Co_x_Ni_1−x_Fe_2_O_4_; x = 0.9/SiO_2_/TiO_2_ nanocomposite.

To reveal the optical characteristics of the synthesized nanocomposite and bare C-dots, UV–Vis. analysis was carried out, as shown in Fig. 3. A strong absorption peak at (299 nm) was recorded for the prepared Co_x_Ni_1−x_Fe_2_O_4_; x = 0.9/SiO_2_/TiO_2_/C-dots nanocomposite. While a C-dots absorption peak was recorded at (256 nm), this could be attributed to π → π * transitions of carbon^66^. It is worth mentioning that the loading of C-dots resulted in a change of the nanocomposite absorption from (365 nm) for the previously-prepared Co_x_Ni_1−x_Fe_2_O_4_; x = 0.9/SiO_2_/TiO_2_ nanocomposite to (299 nm) for the newly-prepared C-dots-loaded Co_x_Ni_1−x_Fe_2_O_4_; x = 0.9/SiO_2_/TiO_2_ nanocomposite^48^. This shift could be attributed to the existence of the new transition electronic bands due to loading with carbon^67,68^. While, band gap energy of the prepared nanocomposite was determined using Tauc’s equation as follows (Eq. 2):

2 $$\upalpha {\text{h}}\upsilon = {\text{A}}\left( {{\text{h}}\upsilon - {\text{Eg}}} \right)^{n}$$

where (α) denotes the absorption coefficient, (hυ) represents photon energy, (A and h) are constants, (Eg) is the band gap energy and (n) is a constant depends on the type of electronic transition and n = 1/2, in case of indirect band gap semiconductors. By plotting a graph of (αhυ) 1/2 versus hυ, linear region’s extrapolation gives the value of band gap energy (Eg) as shown in supplementary Fig. 2. The calculated band gap energy of the synthesized nanocomposite has been found to be (3.35 eV).

#### Surface area and pore size distribution analysis

**Figure 4:**
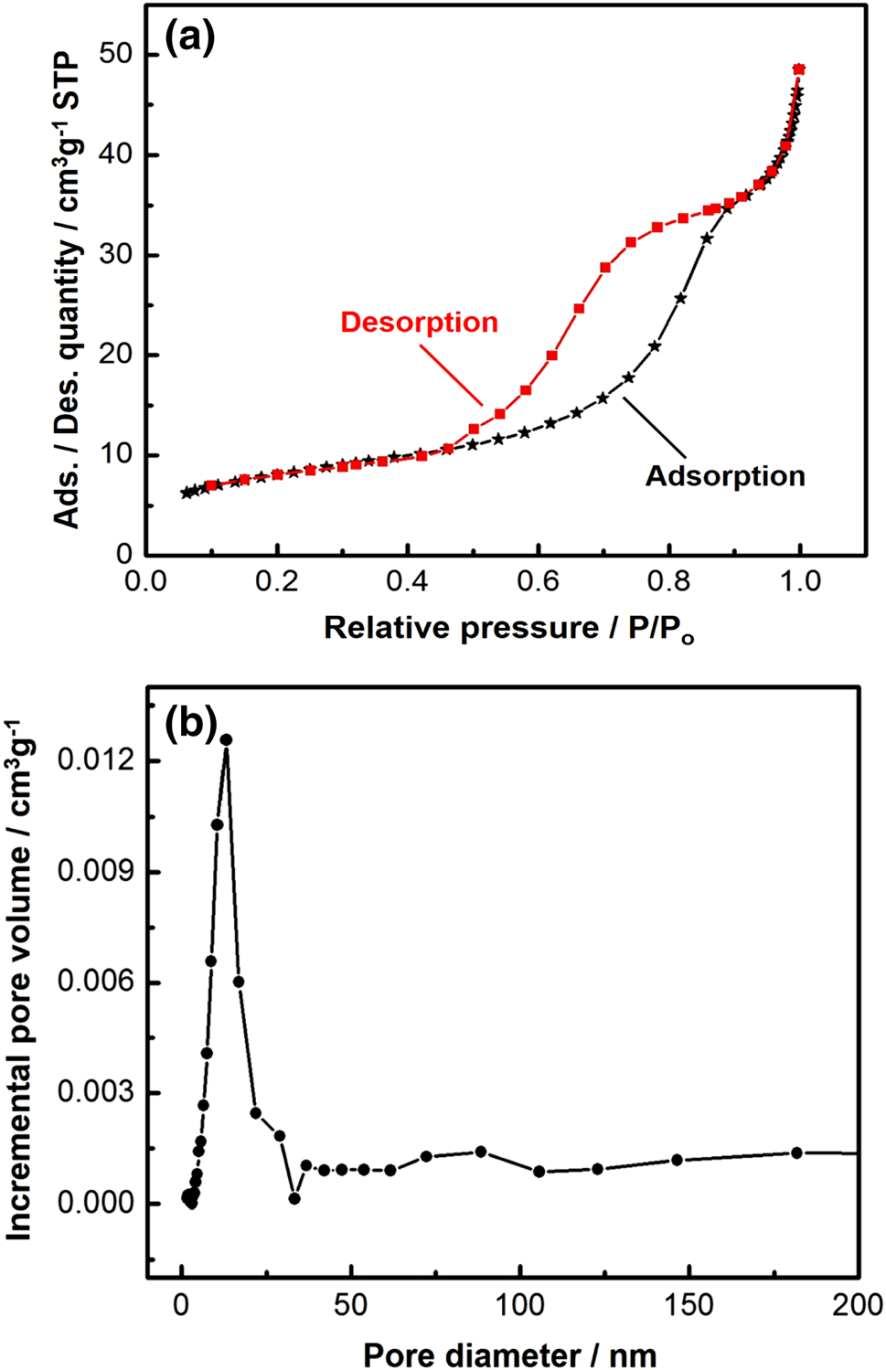
(**a**) N_2_ Adsorption–desorption isotherm of the prepared Co_x_Ni_1−x_Fe_2_O_4_; x = 0.9/SiO_2_/TiO_2_/C-dots nanocomposite; (**b**) Pore size distribution.

N_2_ adsorption–desorption isotherm and pore size distribution of the prepared nanocomposite are shown in Fig. 4a,b. According to the IUPAC classification, the obtained isotherm was of type (IV), indicating the presence of mesopores. The uptake of adsorbate was increased when pores became filled, and an inflection point occurred near the completion of the first monolayer^69–71^. In addition, sharp capillary condensation was recorded at higher pressures (0.95–1), which indicated the presence of macropores^48,72^. The calculated surface area of the prepared nanocomposite was 28.29 ± 0.19 m^2^/g and pore volume was 0.001253 cm^3^/g. Finally, Fig. 4b shows the pore size distribution of the prepared nanocomposite. The prepared nanocomposite possessed unimodal and narrow pore size distribution, with an intense peak at (pore diameter = 13.3 nm), confirming the presence of mesopores.

#### TEM, HR-TEM analysis and average particle size calculation

**Figure 5:**
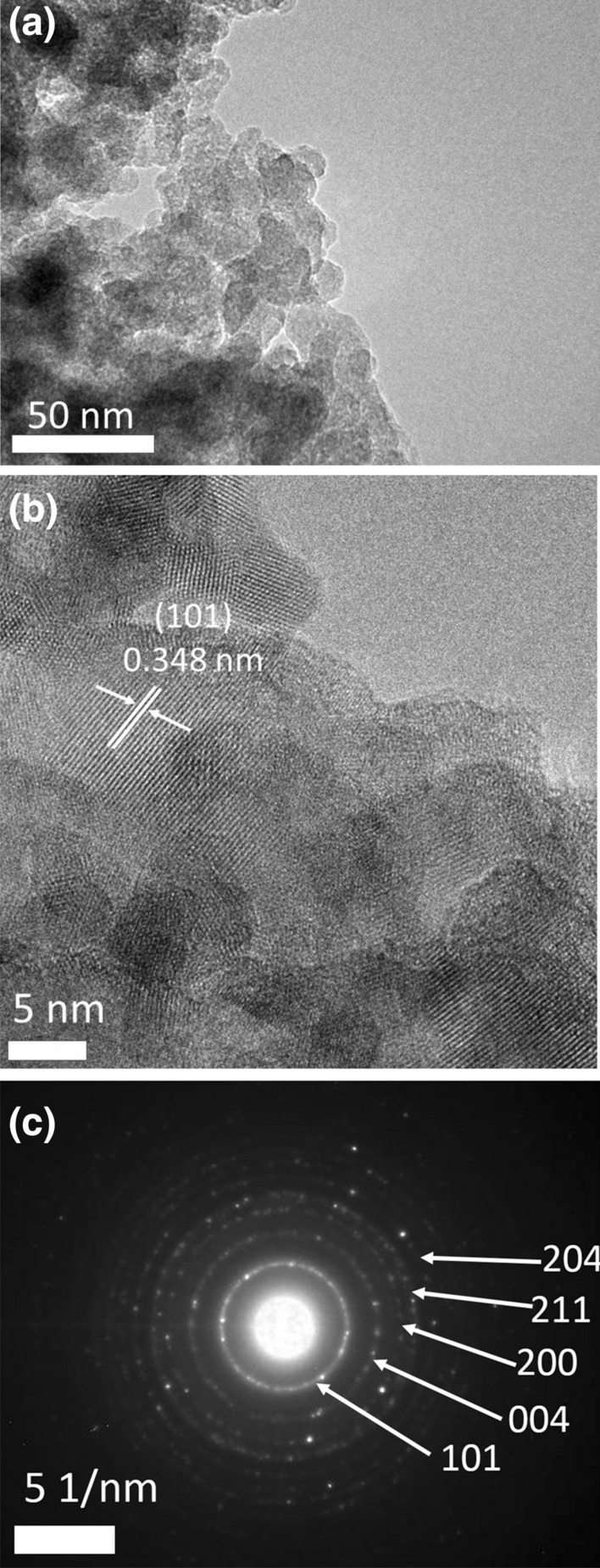
(**a**) TEM image of the prepared Co_x_Ni_1−x_Fe_2_O_4_; x = 0.9/SiO_2_/TiO_2_/C-dots nanocomposite; (**b**) HR-TEM image; (c) SAED pattern.

**Figure 6:**
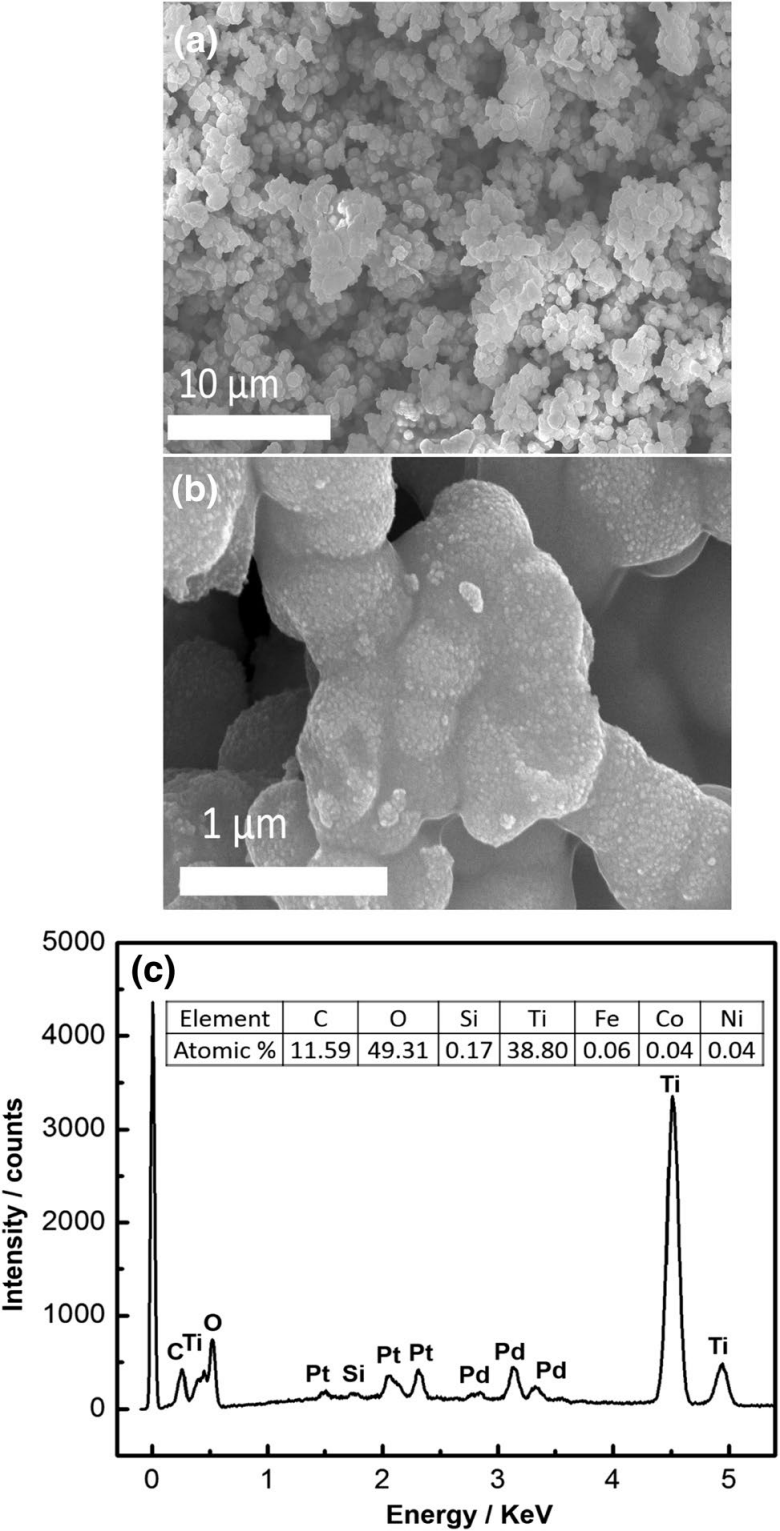
(**a**) SEM image of the prepared Co_x_Ni_1−x_Fe_2_O_4_; x = 0.9/SiO_2_/TiO_2_/C-dots nanocomposite; (**b**) Magnified SEM image; (**c**) EDX pattern with elemental composition.

A TEM image of the prepared nanocomposite is shown in Fig. 5a. The primary particles were agglomerated with high interparticle void content. This structure was in good agreement with the N_2_-gas adsorption–desorption isotherm shown in Fig. 4a, where condensation in interparticle voids clearly-appeared at the high relative pressure region of 0.95–1. In addition, the agglomeration mainly occurred during TEM sample preparation, since the nanocomposite was well-dispersed in water solvent and no precipitation was observed for several hours. The HR-TEM image in Fig. 5b shows that the average particle size was 19 nm. Almost all fringes observed were attributed to anatase TiO_2_ NPs, which can be attributed to the high content and crystallinity of TiO_2,_ as shown in Fig. 6c. The selected area electron diffraction pattern in Fig. 5c also showed only the characteristic rings of anatase TiO_2_. This result was a good match to the recorded XRD pattern in Fig. 2b, where anatase TiO_2_ was the predominant crystal phase of the prepared nanocomposite.

#### SEM and EDX analysis

The external morphology, purity, and the elemental composition of the prepared nanocomposite were studied, as shown in Fig. 6a–c. SEM analysis showed that the prepared nanocomposite had a semi-spherical structure, with a uniform distribution of each layer. EDX analysis revealed the high purity of the prepared nanocomposite, as indicated by the presence of atoms characteristic to each component of it and the absence of foreign atoms that may appear as impurity.

#### Surface bonding and functional groups analysis; FTIR analysis of the prepared nanocomposite

**Figure 7:**
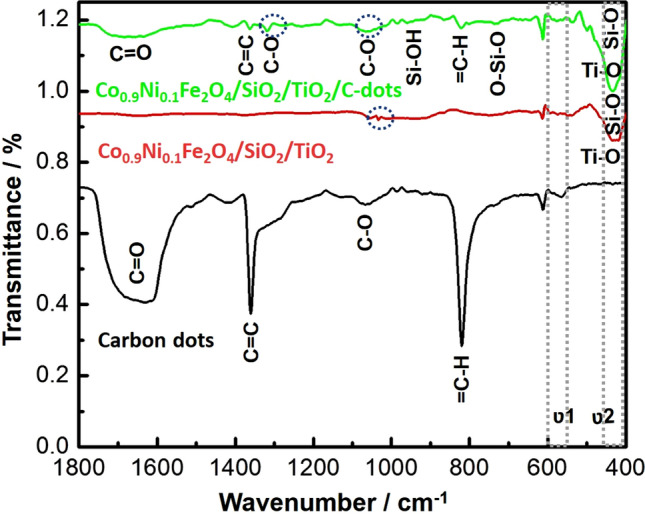
FTIR analysis of the Co_x_Ni_1−x_Fe_2_O_4_; x = 0.9/SiO_2_/TiO_2_/C-dots nanocomposite, bare C-dots and Co_x_Ni_1−x_Fe_2_O_4_; x = 0.9/SiO_2_/TiO_2_ nanocomposite.

Inducing chemical compounds via IR wave’s causes either stretching or bending of these bonds and FT-IR was used to identify the functional groups and define the molecular structure of the studied nanocomposite. The FT-IR investigation was directed to determine the interaction between Co_x_Ni_1−x_Fe_2_O_4_; x = 0.9/SiO_2_/TiO_2_/nanocomposite and the synthesized C-dots (Fig. 7). The observed bands around 455.7 cm^−1^ (in both Co_x_Ni_1−x_Fe_2_O_4_; x = 0.9/SiO_2_/TiO_2_/nanocomposite and Co_x_Ni_1−x_Fe_2_O_4_; x = 0.9/SiO_2_/TiO_2_/C-dots nanocomposite) were assigned to Ti–O stretching vibration^73^. The presence of free silanol (Si–OH) groups on surface was detected at 920.25 cm^−1^^74,75^. In addition, the symmetrical tension of O–Si–O appeared at 789.21 cm^−1^ while the bending of Si–O could be seen at 455.7 cm^−1^^76^. It is generally known that the spinel ferrites exhibit two FTIR active bands, designated as υ_1_ and υ_2_. The ‘υ_1_’ was observed at the range (550–600 cm^−1^) and ‘υ_2_’ was recorded at the range (350–450 cm^−1^). These two bands refer to the stretching of metal ions and oxygen bonds in the tetrahedral and octahedral sites, respectively^77^. Further, the cubic spinal phase of the present samples was successfully-formed^78–81^, as shown in Fig. 7.

The peaks at wavenumber 1155 cm^−1^ were attributed to a bond formation during the synthesis of cobalt nickel ferrite. The stretching of O–H bands can be seen around 1561 cm^−1^. It is clear that the structure remained in the cubic spinel phase even after the substitution of metals on ferrite nanostructures.

It should be noted that, in the FTIR spectrum of the synthesized C-dots, the stretching vibration band of C=O was observed at 1633.3 cm^−1^ (C-dots) and at 1675.0 cm^−1^ (Co_x_Ni_1−x_Fe_2_O_4_; x = 0.9/SiO_2_/TiO_2_/C-dots nanocomposite), and the stretching vibration bands of C–O was detected at 1099 cm^−1^ (C-dots) and at 1089.2 cm^−1^ (Co_x_Ni_1−x_Fe_2_O_4_; x = 0.9/SiO_2_/TiO_2_/C-dots nanocomposite)^82^. Moreover, the obvious two sharp peaks, at 1359 cm^−1^ (C-dots), 1362 cm^−1^ (Co_x_Ni_1−x_Fe_2_O_4_; x = 0.9/SiO_2_/TiO_2_/C-dots nanocomposite) and 818.2 cm^−1^ (C-dots), 824.12 cm^−1^ (Co_x_Ni_1−x_Fe_2_O_4_; x = 0.9/SiO_2_/TiO_2_/C-dots nanocomposite), were associated with the stretching and bending vibrations of C=C, and =C–H, respectively, which suggested the presence of alkyl groups^83^.

Finally, one distinct peak, located at 1319.7 cm^−1^ in Co_x_Ni_1−x_Fe_2_O_4_; x = 0.9/SiO_2_/TiO_2_/C-dots nanocomposite, was not detected in C-dots spectrum. It may have corresponded to the C–O functional group, which suggested the conjugation of C atom with O atoms (from SiO_2_ and/or TiO_2_; inner and outer nanocomposite layers) by a covalent bond. This C-O stretching vibration represented the conjugation of C-dots on the surface of the synthesized Co_x_Ni_1−x_Fe_2_O_4_; x = 0.9/SiO_2_/TiO_2_ nanocomposite^84,85^. Our FTIR results were similar to some recently-published research studies^86–89^.

### Photocatalytic activity of Co_x_Ni_***1−x***_Fe_***2***_O_4_; x =0.9/SiO_2_/TiO_2_/C-dots nanocomposite

**Figure 8:**
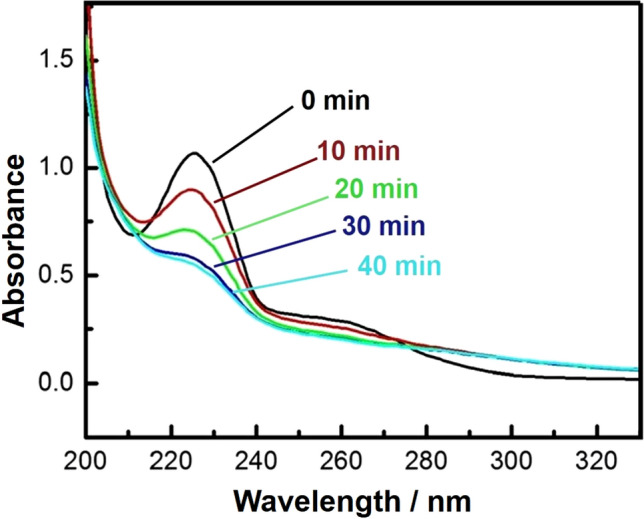
UV–Vis. spectra of chloramine-T solution after photodegradation test for 0–40 min (10 mg of nanocomposite, 50 ml chloramine-T solution, Temp. = 25 °C and pH 7).

**Figure 9:**
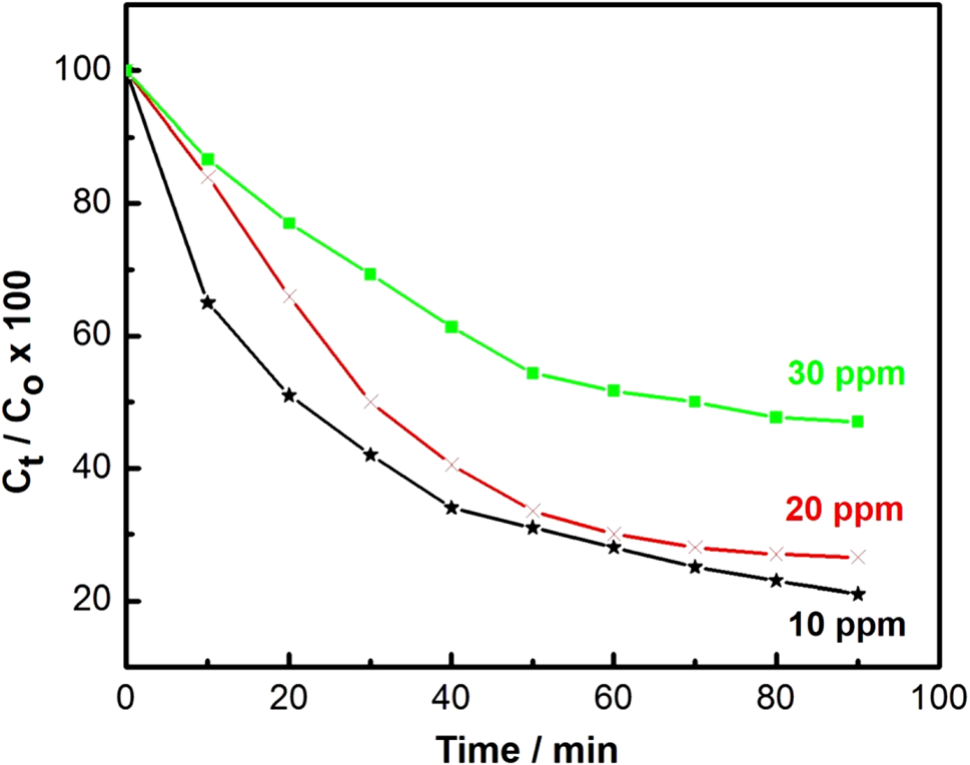
Effect of initial concentration of chloramine-T on the degradation efficiency (10 mg of nanocomposite, 50 ml chloramine-T solution, Temp. = 25 °C and pH = 7).

**Figure 10:**
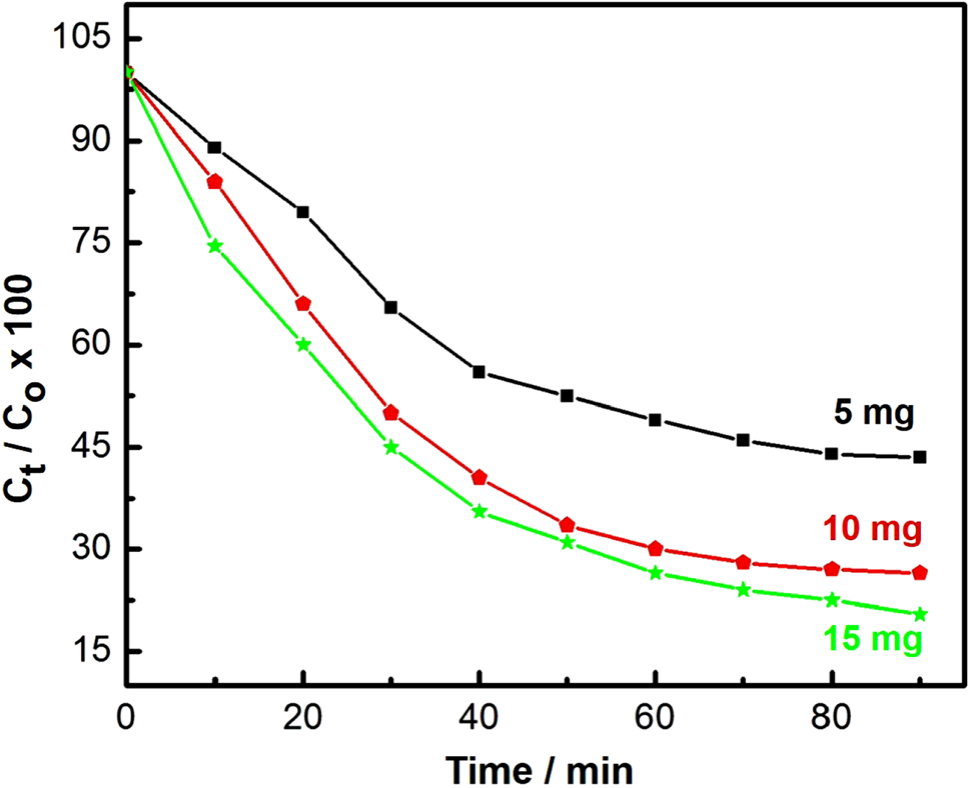
Effect of the photocatalyst dose on the degreadation efficency of chloramine-T (50 ml chloramine-T solution (20 mg/l), Temp. = 25 °C and pH 7).

The photocatalytic activity of the synthesized C-dots-loaded nanocomposite was evaluated via the photocatalytic degradation of an aqueous solution of chloramine-T trihydrate under UV-light irradiation. Upon increasing the UV-irradiation period, the strong absorption bands of chloramine-T recorded at 225 nm (the maximum absorbance wavelength (λ_max_) for the chloramine-T), reduced continuously, and the removal of chloramine-T solution reached about 80% after 90 min of UV-light irradiation, as shown in Figs. 8, 9, and 10).

#### Effect of chloramine-T initial concentration on the degradation efficiency

Degradation efficiency with an irradiation time of chloramine-T via 10 mg of the prepared nanocomposite in (50 ml) chloramine-T solution of initial concentrations (10, 20 and 30 mg/l) is illustrated in Fig. 9. The color of the solution turned from a turbid white to nearly transparent at the end of the decomposition experiment, with approximately 80% removal after 90 min (Figs. 9, 10). Our results showed that the degradation efficiency of chloramine-T is inversely-proportional to its initial concentration. The decomposed percentage of chloramine-T was measured by using C_t_/C_o_ × 100, where C_t_ and C_o_ are the remaining and initial concentrations of chloramine-T, respectively.

#### Effect of the nanocompositedose on degradation efficiency

The influence of a nanocomposite dose on the photodegradation of chloramine-T under UV-light was studied by varying the amount of the prepared photocatalyst between 5 and 20 mg against a fixed concentration of chloramine-T (20 mg/l), as shown in Fig. 10. The results showed that by increasing the amount of the employed photocatalyst from (5 to 20 mg), a decrease in the value of C_t_/C_o_ × 100 was observed from 40 to 20, respectively. The results also indicated an increase in the degradation efficiency upon increasing the photocatalyst dose from (5 to 20 mg). The observed increase in degradation efficiency with increasing the amount of the photocatalyst in the reaction could be attributed to the increase in the available active area or active sites of the photocatalyst to volume ratio of chloramine-T solution^90,91^.

**Figure 11:**
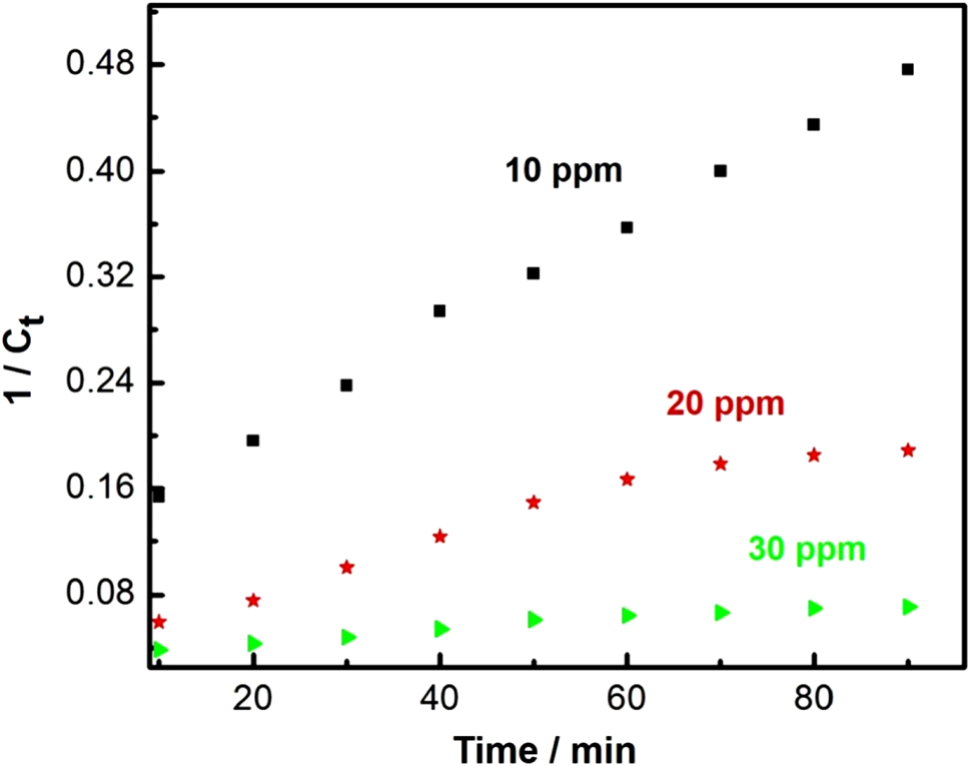
Pseudo second order kinetics of chloramine-T degradation (10 mg of nanocomposite, 50 ml chloramine-T solution, Temp = 25 °C and pH 7).

**Figure 12:**
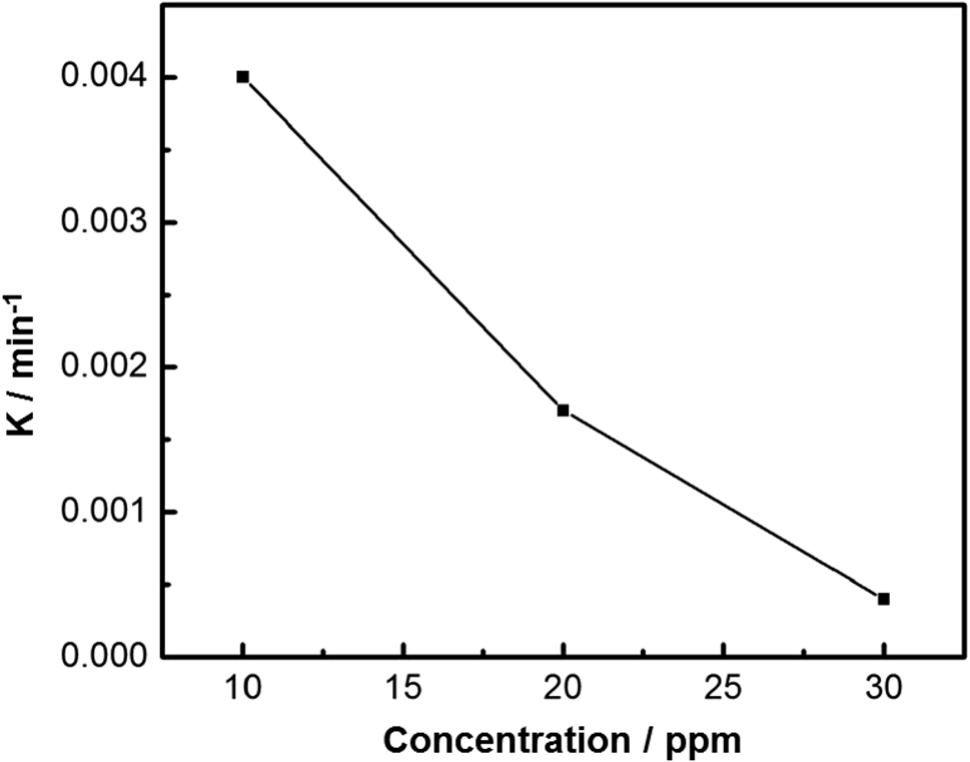
Apparent pseudo second order rate constant vs initial concentration of chloramine-T.

While Fig. 11 shows a plot of 1/C_t_ against time, which gives a straight-line with intercept equal to 1/C_0_ and slope *k*. According to the values of *R*^2^ > 99.5, the reactions of chloramine-T degradation with the prepared nanocomposite followed pseudo second-order reaction kinetics. Moreover, as indicated in Fig. 12, there is a clear inverse dependence of the apparent pseudo second-order rate constant on the initial concentration of chloramine-T.

#### Effect of pH value on the photodegradation of chloramine-T

**Figure 13:**
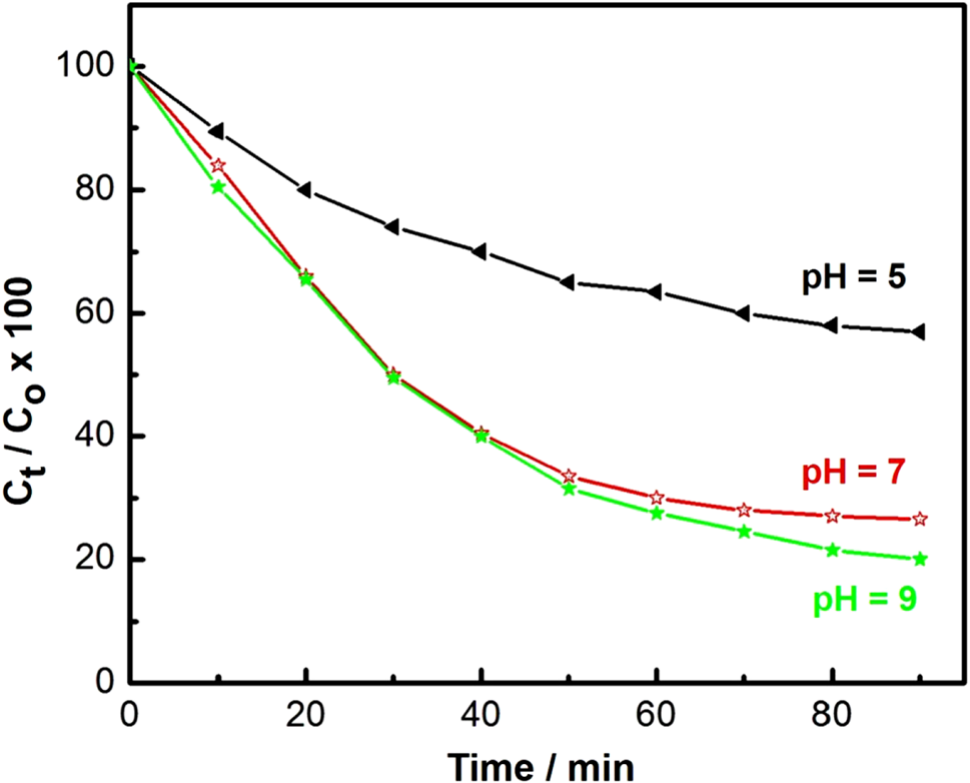
Effect of pH on the degradation of chloramine-T (10 mg nanocomposite, 50 ml chloramine-T (20 mg/l) and 100 min UV-irradiation).

In this part, the role of reaction pH on the photocatalytic degradation of chloramine-T was studied in the pH range from (5 to 9) at room temperature (25 ± 2 °C). The initial pH of the chloramine-T solution was set before UV-irradiation and it was not changed during the experiments. The influence of the initial pH on the photodegradation of chloramine-T under UV-irradiation was investigated, and the results are shown in Fig. 13.

The photodegradation of chloramine-T was enhanced by increasing the pH. It is well known that, the photocatalytic degradation of chloramine-T is a complicated process, which started with the adsorption on the nanocomposite surface^92^. The photocatalytic performance of the prepared nanocomposite could be attributed to the surface electrical properties, due to the different interlayer anions. The superior surface potential of the nanocomposite facilitated chloramine-T adsorption, which is helpful in promoting the transfer of light-generated charge carriers to the photocatalyst surface^93^.

The initial pH of the solution is one of the most remarkable parameters controlling the photocatalytic process and can affect the surface charge nature of the photocatalyst and the extent of agglomeration and its stability^47,93,94^. Moreover, pH manipulates a significant role in the reaction mechanisms that can lead to chloramine-T degradation.

The photodegradation mechanisms affected by varying the pH values include hydroxyl radical attack, explicit oxidation by the positive holes in the valence band, and explicit reduction by the electrons in the conduction band. In the presence of a photocatalyst, it is assumed that photocatalytic degradation is likely to happen due to the formation of electron–hole pairs on the exterior of the employed photocatalyst, due to UV-irradiation. Oxidative potential of holes either reacts with the–OH groups to form hydroxyl radicals or oxidizes the reactive chloramine-T to form a degradation product^95^. The reactions of chloramine-T and the employed photocatalyst can be summarized as follows (Eqs. 3–6).

2 $${\text{C}} - {\text{dots }}\;{\text{loaded }}\;{\text{composite}} + {\text{h}}\nu { } \to {\text{nanocomposite }}\left( {{\text{e}}_{{{\text{CB}}}}^{ - } + {\text{ h}}_{{{\text{VB}}}}^{ + } } \right){ }$$

3 $${\text{h}}_{{{\text{VB}}}}^{ + } + {\text{Chloramine}} - {\text{T}} \to {\text{Chloramine}} - {\text{T}}^{ \cdot + } { }\left( {{\text{Oxidation}}\;{\text{of }}\;{\text{the }}\;{\text{compound}}} \right){ }$$

Or

4 $${\text{h}}_{{{\text{VB}}}}^{ + } + {\text{OH}}^{ - } \to {\text{OH}}^{ \cdot } { }$$

5 $${\text{OH}}^{ \cdot } + {\text{chloramine}} - {\text{T}} \to \left( {{\text{Degredation }}\;{\text{product}}} \right){ }$$

Interestingly, the concentration of OH^**·**^ radicals is relatively-higher at higher pH values (alkaline medium), and this may also be another reason for the increase in photodegradation of strong alkaline media. In addition, the high pH value (in alkaline media) is beneficial to the formation of OH^**·**^ radicals during the reaction between dissolved oxygen and excited state electrons, which makes the degradation of chloramine-T noteworthy^96^, while, at low pH values, a decrease in photodegradation efficiency is noticed that may be attributed to the instability of the prepared nanocomposite through a cathodic dislocation of the valence band position, which gives rise to a weakening of the oxidation capability of the holes. In conclusion, the initial reaction pH has an influence on the surface charge of the catalyst and the adsorption characteristics of ions^47,95^.

**Figure 14:**
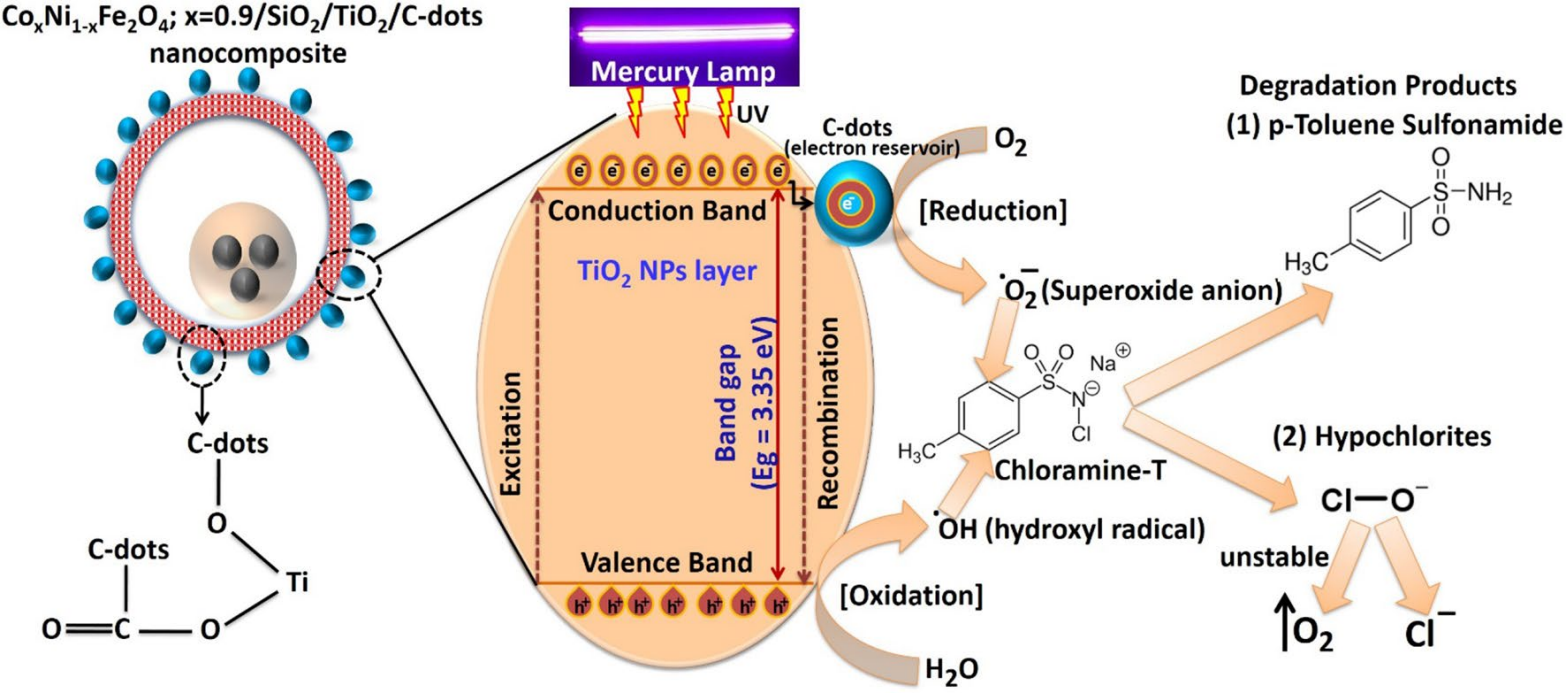
Proposed mechanism of photocatalytic degradation of chloramine-T with the prepared nanocomposite.

The proposed mechanism of interaction between the prepared nanocomposite and chloramine-T is shown in Fig. 14. Upon UV-light excitation of TiO_2_ layer, charge carriers will be photogenerated and redox reactions will be initiated. Then, the generated free radicals (such as OH^**·**^ And O_2_^**·**−^ ) will degrade chloramine-T into two potential products, p-touluene sulfonamide and hypochlorites that can be easily dissociated into O_2_ and Cl^-^ ions. Since, there are no published reports about the degradation of chloramine-T till the moment, more investigations via high-performance liquid chromatography (HPLC) and gas chromatography-mass spectrometry (GC–MS) are required to analyze with more details the degradation products of chloramine-T.

### In vitro antimicrobial activity of the synthesized Co_x_Ni_1−x_Fe_2_O_4_; x = 0.9/SiO_2_/TiO_2_/C-dots nanocomposite

**Figure 15:**
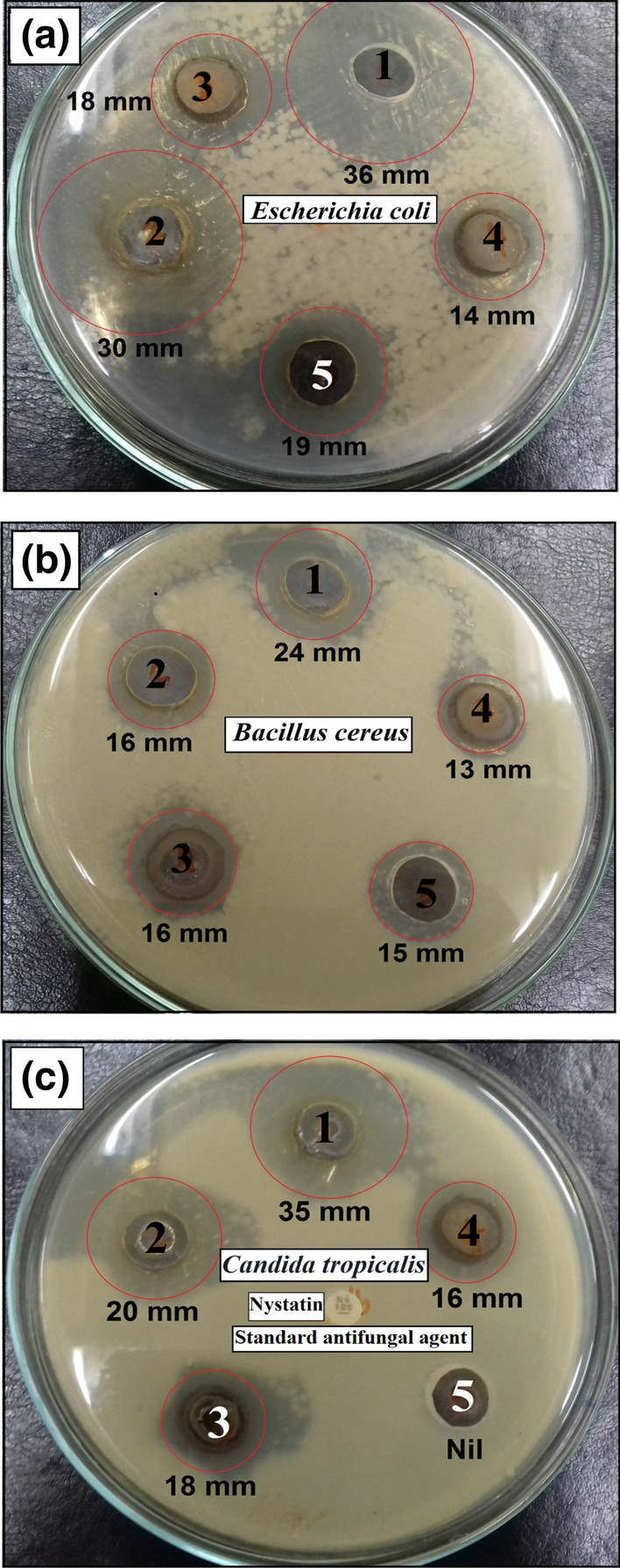
Antibacterial and antifungal activities of bare C-dots and Co_x_Ni_1−x_Fe_2_O_4_; x = 0.9/SiO_2_/TiO_2_/C-dots nanocomposite on: (**a**) *Escherichia coli*, (**b**) *Bacillus cereus* and (**c**) *Candida tropicalis* measured as ZOI (mm). 1 = Co_x_Ni_1−x_Fe_2_O_4_; x = 0.9/SiO_2_/TiO_2_/C-dots nanocomposite (15 µg/ml), 2 = Co_x_Ni_1−x_Fe_2_O_4_; x = 0.9/SiO_2_/TiO_2_/C-dots nanocomposite (10 µg/ml), 3 = bare C-dots (15 µg/ml), 4 = bare C-dots (10 µg/ml) and 5 = Amoxicillin/Clavulanic Acid (100 µg/ml, standard antibacterial agent).

Agar diffusion technique was used to test the antimicrobial potential of the prepared nanocomposite (screening procedure). C-dots-loaded nanocomposite (15 µg/ml) showed comparatively-higher antimicrobial potential against all examined bacteria and *Candida species* compared with C-dots. Screening data verified that the fabricated nanocomposite possessed predominant antibacterial efficacy against *E. coli* (36 mm ZOI, Fig. 15a), *P. aeruginosa* (33 mm ZOI) and *B. cereus* (24 mm ZOI, Fig. 15b) as seen in Table 1. Interestingly, the synthesized C-dots loaded nanocomposite exhibited more effective antimicrobial capacities than bare C-dots and other conventional antimicrobial agents (AMC).

**Table 1:** Antimicrobial activities of bare C-dots and Co_x_Ni_1−x_Fe_2_O_4_; x = 0.9/SiO_2_/TiO_2_/C-dots nanocomposite, against multi-drug-resistant (MDR) bacteria and pathogenic *Candida species*, measured as ZOI (mm) and MIC (μg/ml).

Pathogenic microbes	ZOI of bare c-dots (10 µg/ml) (mm)	ZOI of bare c-dots (15 µg/ml) (mm)	MIC of bare c-dots (µg/ml)	ZOI of CoxNi_1−x_Fe_2_O_4_; x = 0.9/SiO_2_/TiO_2_/C-dots (10 µg/ml) (mm)	ZOI of CoxNi_1−x_Fe_2_O_4_; x = 0.9/SiO_2_/TiO_2_/C-dots (15 µg/ml) (mm)	MIC of CoxNi_1−x_Fe_2_O_4_; x = 0.9/SiO_2_/TiO_2_/C-dots (µg/ml)	AMC or NS
*Escherichia coli*	14^d^ ± 0.2516	18^d^ ± 0.2516	1.562	30^e^ ± 0.1000	36^g^ ± 0.2516	0.024	19^c^ ± 0.2516
*Pseudomonas aeruginosa*	13^c^ ± 0.2081	14^b^ ± 0.1154	1.562	21^d^ ± 0.3000	33^e^ ± 0.4725	0.195	10^a^ ± 0.1527
*Staphylococcus aureus*; MRSA	9^a^ ± 0.4041	10^a^ ± 0.5291	12.5	11^a^ ± 0.2309	12^a^ ± 0.4725	6.25	Nil
*Bacillus cereus*	13^c^ ± 0.4509	16^c^ ± 0.1000	0.751	16^b^ ± 0.4506	24^c^ ± 0.1154	0.390	15^b^ ± 0.3214
*Klebsiella pneumoniae*	9^a^ ± 0.4509	10^a^ ± 0.2516	6.25	16^b^ ± 0.4041	18^b^ ± 0.1154	3.125	Nil
*Candida albicans*	12^b^ ± 0.4041	16^c^ ± 0.2516	3.125	16^b^ ± 0.1732	25^d^ ± 0.2000	0.781	Nil
*Candida tropicalis*	16^e^ ± 0.3214	18^e^ ± 0.2516	0.781	20^c^ ± 0.3000	35^f^ ± 0.3055	0.097	Nil
LSD	0.76667	1.50000	–	4.03333	0.96667	–	–

Values are presented as means ± SD (n = 3). Data within the groups were analyzed using one-way analysis of variance (ANOVA) followed by superscript letters (a–g) Duncan’s multiple range test (DMRT), *LSD* least significant difference.

Nil means that no ZOI was measured. *AMC* amoxicillin/clavulanic acid (standard antibacterial agent). *NS* Nystatin (standard antifungal agent).

Our previously-prepared Co_x_Ni_1−x_Fe_2_O_4_ x = 0.9 /SiO_2_/TiO_2_; nanocomposite^47^ exhibited antibacterial action of (16 mm, ZOI) against *E. coli* and an antifungal potential against *C. albicans* of (10 mm, ZOI). Interestingly, by making a comparison, we observed an enhanced antibacterial activity of C-dots-loaded nanocomposite (Fig. 15, Table 1) suggesting the possibility of synergistic potential between Co_x_Ni_1−x_Fe_2_O_4_/SiO_2_/TiO_2_; x = 0.9 nanocomposite and C-dots.

It was also noted that the prepared nanocomposites were more effective against Gram-negative than Gram-positive bacteria. One possible cause is in how the bacterial cell walls are constructed, as cell walls of Gram-negative species consist primarily of films (thin layers) of peptidoglycans, lipopolysaccharides, and lipids, while, cell walls of Gram-positive species have thick arrangements of peptidoglycans^97^.

The prepared C-dots-loaded nanocomposite can be used as a powerful antifungal agent, as it possesses an extraordinary antifungal potency against *C. tropicalis* (35 mm ZOI, Fig. 15c) and *C. albicans* (28 mm ZOI) as presented in Table 1.

The MIC values of bare C-dots and C-dots-loaded nanocomposite against all tested pathogenic bacteria and *Candida* sp. were in the range of 6.25 to 0.024 μg/ml, as shown in Table 1. The synthesized nanocomposite possessed MIC values of about 0.024 μg/ml against *E. coli*, 0.097 μg/ml against *C. tropicalis*, and 0.390 μg/ml against *B. cereus.*

Surprisingly, by comparing these results with those in our previously-published paper^47^, the synthesized Co_x_Ni_1−x_Fe_2_O_4_; x = 0.9 /SiO_2_/TiO_2_ nanocomposite possessed MIC values of (3.12 µg/ml) against *E. coli* and (12.5 µg/ml) against *C. albicans*. However, the newly-prepared C-dots loaded nanocomposite showed more promising MIC results, of about (0.024 µg/ml against *E. coli*) and (0.781 µg/ml ZOI against *C. albicans*), suggesting it had good antimicrobial abilities at very low concentrations.

Interestingly, there is a correlation between the physical characteristics (surface area) of the prepared nanocomposite and its recorded antimicrobial capabilities. The measured surface area of the C-dots-loaded nanocomposite was (28.29 ± 0.19 m^2^/g) with a unimodal and narrow pore size distribution, with an average pore diameter of (13.3 nm) and an average pore volume of (0.001253 cm^3^/g). The prepared nanocomposite possessed two classes of pores in its outer shell (TiO_2_ NPs), mesoporous and macropores^98^. This surface area and pore size distribution expanded its connection areas (active sites) to absorb more microbial cells (diameter of *E. coli* is 0.25 µm). These physical features were significant in enhancing its antimicrobial potency at a low concentration (0.024 µg/ml) against all tested pathogenic bacteria and *Candida* species.

### Effect of UV-irradiation on the antimicrobial potential of Co_x_Ni_1−x_Fe_2_O_4_; x = 0.9/SiO_2_/TiO_2_ /C-dots nanocomposite in liquid media

**Figure 16:**
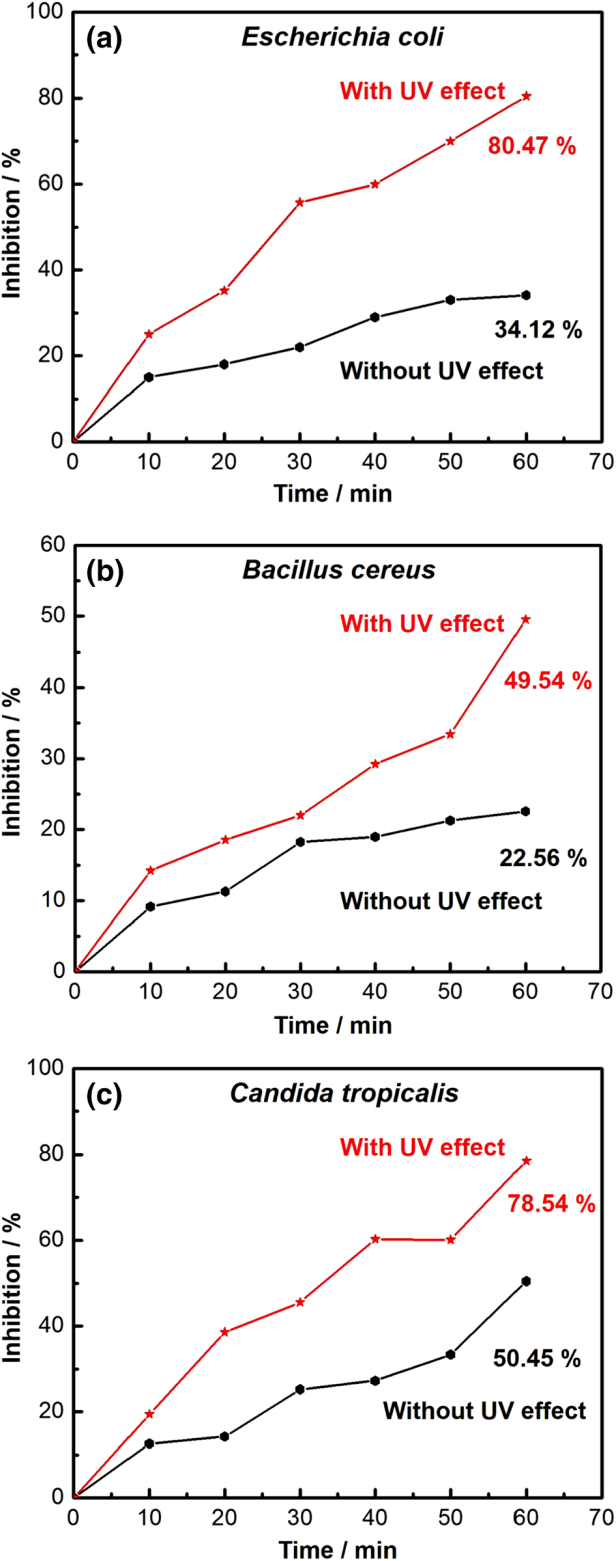
Antimicrobial activity of UV-irradiated Co_x_Ni_1−x_Fe_2_O_4_; x = 0.9/SiO_2_/TiO_2_/C-dots nanocomposite against different pathogenic microbes: (**a**) *Escherichia coli*, (**b**) *Bacillus cereus* and (**c**) *Candida tropicalis.*

Comparative study of the repression percentage of *E. coli*, *B. cereus*, and *C. tropicalis* by non-irradiated and UV-irradiated nanocomposite was conducted and is shown in Fig. 16.

The restraint percentage of the examined pathogens due to nanocomposite treatment decreased as a function of time, suggesting that it maintained effective antimicrobial capabilities against the colonies of *E. coli*, *B. cereus*, and *C. tropicalis*. as shown in Fig. 16a–c.

Interestingly, the UV-irradiated nanocomposite showed higher antimicrobial potential compared with the non-irradiated, as shown in Fig. 16.

The highest recorded hindrance percentages of non-irradiated and UV-irradiated nanocomposites against *E. coli* were 34.12% and 80.47%, respectively. Inhibition percentages were 22.56% and 49.54% for *B. cereus* and 50.45% and 78.54% for *C. tropicalis* after 60 min of UV-irradiation (practice time) as shown by non-irradiated and UV-irradiated nanocomposites, respectively.

The effect of UV-irradiation on the nanocomposite can be explained by the photo-created reactive oxygen species (ROS), which can disintegrate bacterial cells. The observed antimicrobial capabilities were also due to the effective UV-absorption by the synthesized nanocomposite. OH radicals can be also produced by irradiating the nanocomposite with UV. Due to the electron shift within the microbial cells and the nanocomposite, OH radicals can damage bacterial cells by reducing co-enzyme contents^99^.

In addition, metal oxides (MOs), like TiO_2_ (the composite’s external layer), possess positive charges in slightly-acidic media, while microbes have negative charges. This creates an electromagnetic attraction between microbes and MOs, resulting in microbial cell oxidization and consequent damage^100^. Moreover, nanomaterials can damage cellular proteins and DNA by binding with electron-donating constructions such as thiols, carbohydrates, indoles, hydroxyls, and amides. Additionally, they can induce cracks in the cell walls of bacteria, causing extensive permeability and cell death^101^. We previously-reported that our Co_x_Ni_1−x_Fe_2_O_4_; x = 0.9 /SiO_2_/TiO_2_ nanocomposite had a negative charge in neutral media, but tested microbes media is slightly-acidic (pH = 6), which can change the outside charge of the nanocomposite to positive, which is in a good agreement with our recorded results.

Further, our previous Co_x_Ni_1−x_Fe_2_O_4_; x = 0.9/ SiO_2_/ TiO_2_ nanocomposite^47^ displayed an inhibition % against *E. coli* of about 70.45% after UV-activation, while the newly-synthesized Co_x_Ni_1−x_Fe_2_O_4_; x = 0.9/SiO_2_/TiO_2_/ C-dots nanocomposite showed an inhibition % of 80.47% (Fig. 16a). In addition, its repression to *Candida* species reached (78.54%; Fig. 16c) compared with only 50.85% for the previous Co_x_Ni_1−x_Fe_2_O_4_; x = 0.9/SiO_2_/TiO_2_ nanocomposite^47^.

### Antibiofilm potential of Co_x_Ni_1−x_Fe_2_O_4_; x = 0.9/SiO_2_/TiO_2_/C-dots nanocomposite

Biofilm formation is popular in various exopolysaccharide-producing pathogenic microorganisms^59,102^. Biofilms are formed by the tested pathogenic bacteria and yeast with and without nanocomposite treatment was evaluated by using test tubes process^103^.

**Figure 17:**
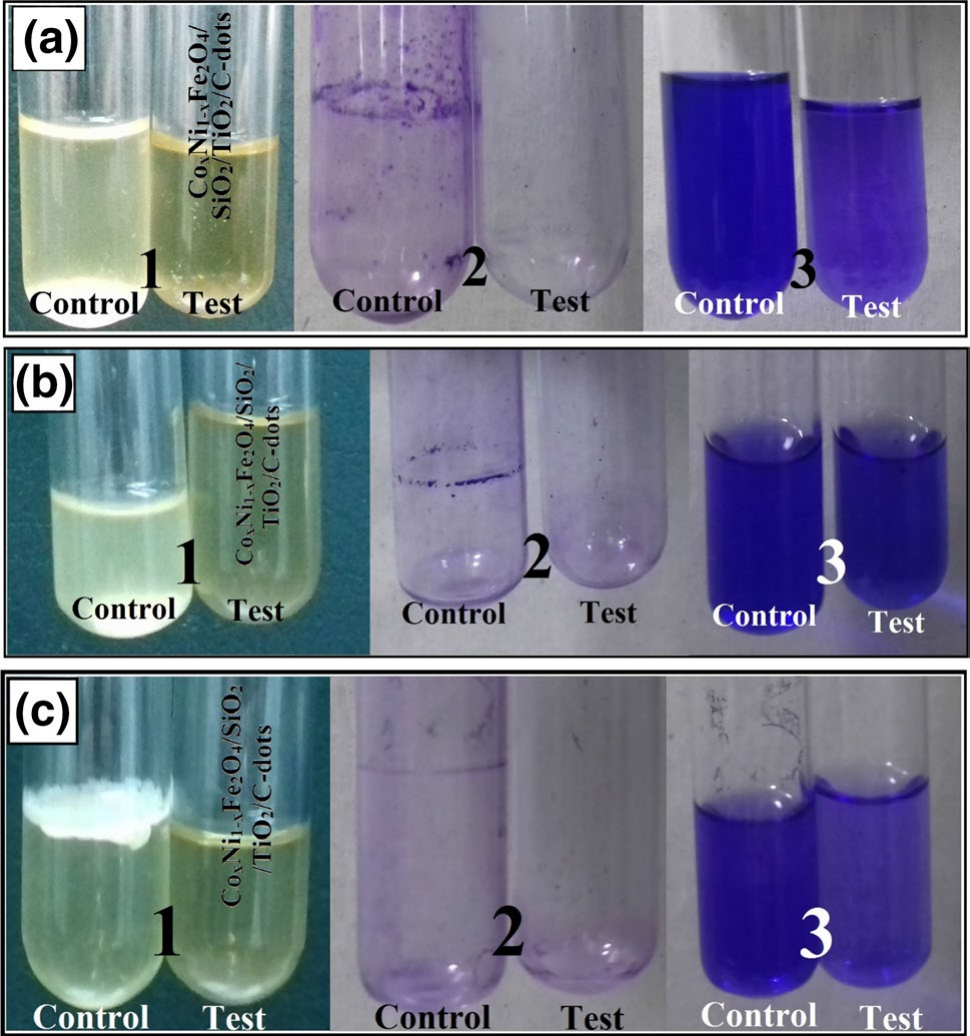
Antibiofilm activity of Co_x_Ni_1−x_Fe_2_O_4_; x = 0.9/SiO_2_/TiO_2_/C-dots nanocomposite. (15 µg/ml) using tube method against (**a**) *Escherichia coli,* (**b**) *Bacillus cereus* and (**c**) *Candida tropicalis*, showing (1) Growth of the bacterial and yeast cells and biofilm formation (rings) without nanocomposite treatment and the inhibition of bacterial and yeast growth after treatment, (2) Staining of the adherent bacterial and yeast cells with crystal violet and (3) Removal and dissolution of the adherent bacterial and yeast cells by ethanol for semi-quantitative biofilm inhibition determination (as shown in Table 2).

Figure 17a displays the antibiofilm ability of the prepared nanocomposite against *E. coli*. *E. coli* grew without our nanocomposite and shows a clear whitish-yellow matt in the air–liquid interface of the tubes. This matt was completely-connected to the inner wall of the tubes and resembled a blue circle after CV staining. A blue solution was also formed after dissolving the CV-stained circle by absolute ethanol, as shown in Fig. 17a.

On the other hand, the restriction of bacterial rings growth was observed in *E. coli* inoculated with tested nanocomposite (15 µg/ml), and the blue color corresponding to CV-stained bacterial cells was faint, as shownin Fig. 16A. Similar results were observed for *B. cereus* and *C. trobicalis* biofilm suppression, as shown in Figs. 17b,c respectively.

To determine the restraint percentage of bacterial and yeast biofilm, a UV–Vis. spectrophotometer was used (at 570 nm). The optical density (O.D.) was measured after separating the CV-stained bacterial and yeast biofilms through ethanol. Table 2 presents the reduction percentage of the biofilms created by the examined bacteria and yeast strains. The highest suppression percentage was recorded against *E. coli* (93.92%, Fig. 17a), followed by *C. trobicalis* (92.35%, Fig. 17c) and *C. albicans* (66.29%, Table 2) after inoculation with (10 µg/ml) of the prepared nanocomposite.

**Table 2:** Semi-quantitative inhibition of the biofilm formation by non-treated and nanocomposite-treated bacterial and yeast pathogens.

Bacterial and yeast strains	O.D. of crystal violet stain at 570 nm (control)	O.D. of crystal violet stain at 570 nm (treated with Co_x_Ni_1−x_Fe_2_O_4_; x = 0.9/SiO_2_/TiO_2_/C-dots nanocomposite)	Inhibition %
*Escherichia coli*	3.981^g^ ± 0.0206	0.242^a^ ± 0.0216	93.92
*Pseudomonas aeruginosa*	1.254^b^ ± 0.0096	0.615^c^ ± 0.0035	50.35
*Staphylococcus aureus*; MRSA	1.060^a^ ± 0.0055	0.564^b^ ± 0.0020	48.49
*Bacillus cereus*	1.735^c^ ± 0.0291	0.606^c^ ± 0.0025	65.07
*Klebsiella pneumoniae*	2.170^d^ ± 0.0026	1.025^e^ ± 0.0020	52.76
*Candida albicans*	2.225^e^ ± 0.0343	0.750^d^ ± 0.0049	66.29
*Candida tropicalis*	2.904^f^ ± 0.0475	0.222^a^ ± 0.0049	92.35
LSD	0.07633	0.04300	–

Values are presented as means ± SD (n = 3). Data within the groups were analyzed using one-way analysis of variance (ANOVA) followed by superscript letters (a–g) Duncan’s multiple range test (DMRT), *LSD* least significant difference.

In addition, the newly-synthesized Co_x_Ni_1−x_Fe_2_O_4_; x = 0.9/SiO_2_/TiO_2_/C-dots nanocomposite was more active against biofilm creation than the previously-synthesized Co_x_Ni_1−x_Fe_2_O_4_; x = 0.9 /SiO_2_/TiO_2_ nanocomposite^47^. It exhibited an inhibition against biofilm-producing *E. coli* of (93.92%, Table 2), compared with (92.82%) for the Co_x_Ni_1−x_Fe_2_O_4_; x = 0.9/SiO_2_/TiO_2_ nanocomposite. Moreover, it inhibited biofilm production by *C. trobicalis* (92.35%, Table 2) compared with only (77.84%) by the Co_x_Ni_1−x_Fe_2_O_4_; x = 0.9/ SiO_2_/TiO_2_ nanocomposite.

The prepared nanocomposite was used to control the biofilm growth at its adhesion level (known as the initial level)^104^. The change in the inhibitory percentage can be assigned to different factors such as antimicrobial potential, biosorption (due to the large exterior surface area of the nanocomposite), physical properties (size of particles and porosity), attack abilities, and many chemical characteristics managing the interaction of the synthesized nanocomposite and biofilms^103,105^.

It was also observed that the prepared nanocomposite significantly-repressed *E. coli* by more than 98% with 0.024 µg/ml (MIC, Table 1). When the exopolysaccharide construction is inhibited (the essential fragments for biofilm expansion), *E .coli* cannot create its biofilm^59,103^.

To clarify the antibiofilm capabilities of the nanocomposite, we attempted an activity mechanism against *E. coli* and *C. tropicalis* biofilms using SEM/EDX analysis^48,106^. SEM images revealed the shape of bacterial and yeast cells before and after nanocomposite treatment.

**Figure 18:**
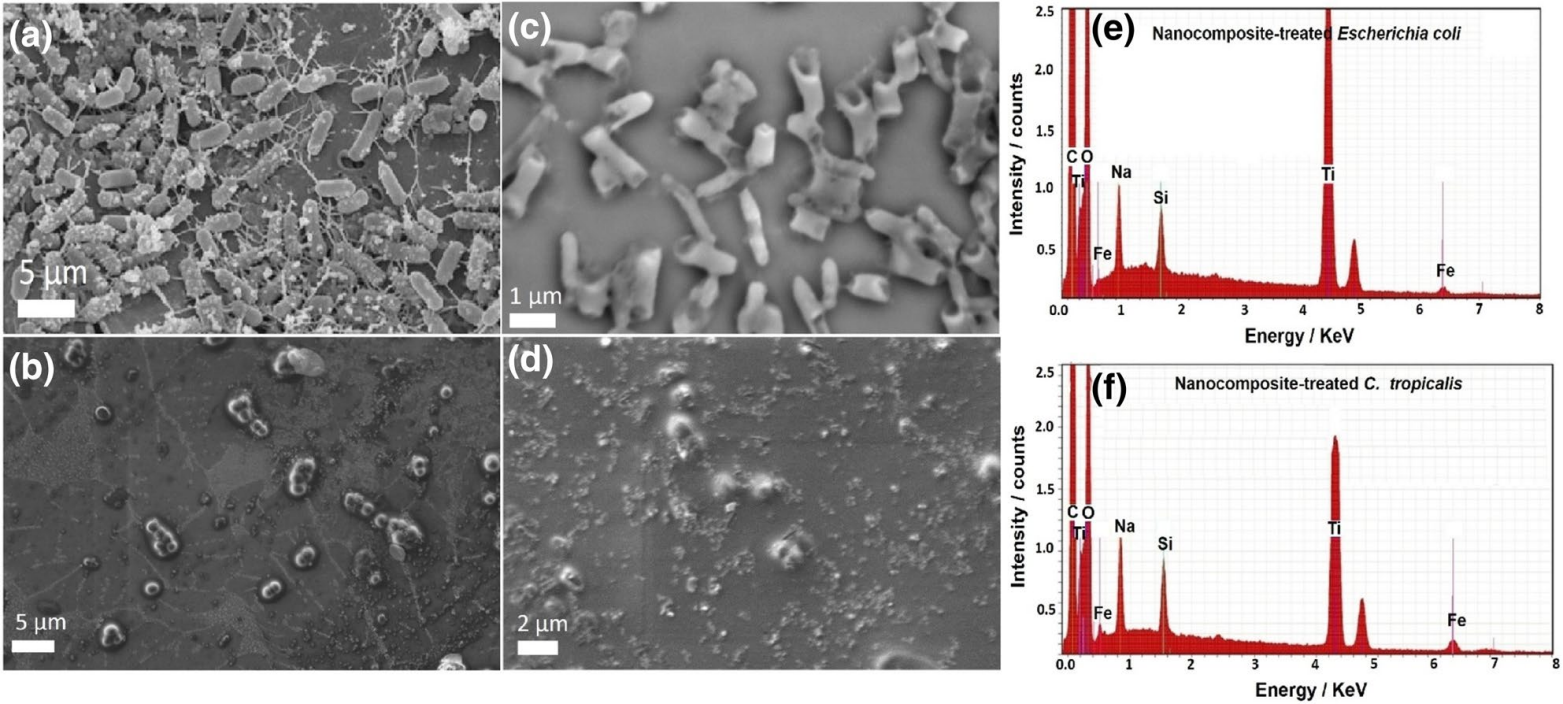
SEM and corresponding EDX elemental analysis of *Escherichia coli* and *Candida trobicalis*: (**a**) Normal bacterial cells (*E. coli*) without nanocomposite treatment, (**b**) Normal yeast cells (*C. tropicalis*) without nanocomposite treatment, (**c**) Depressed and deformed bacterial cell after nanocomposite treatment, (**d**) Depressed and deformed *Candida* cell by nanocomposite treatment showing the complete lysis of *Candida* cell and loss of budding formation, (**e**) Corresponding EDX elemental analysis of the treated *E. coli* cell confirming the cellular internalization of the prepared nanocomposite in *E. coli* cells, and (**f**) Corresponding EDX elemental analysis of the treated *C. tropicalis* cell confirming the cellular internalization of the synthesized nanocomposite in *C. tropicalis* cells.

In the control sample (non-treated bacterial and yeast cells), bacterial and yeast colonies were regularly-grown and exhibited normal cellular shapes with healthy cell surface and concentrated biofilm, as shown in Fig. 18a,b.

After nanocomposite treatment, observable morphological changes were recognized in *E. coli* and *C. tropicalis* cells (Fig. 18c, and d). In addition, an observable lysis of external surface was accompanied by deformations and reductions in the viable number of *E. coli* and *C. tropicalis* cells*.* Furthermore, biofilm development was inhibited. EDX elemental analysis revealed the presence of Ti and Si atoms (atoms of nanocomposite’s outer shells) and C atoms for C-dots at deformation areas and at the outer surface of the treated *E. coli* and *C. tropicalis* cells, confirming the action of the tested nanocomposite, as shown in Fig. 18e, and f.

One possible reason for the powerful activity against the cells of *E. coli* and *C. tropicalis* could be the large surface area (28.29 m^2^/g), which allows for a conventional immobile connection between the negatively-charged bacterial cell walls and the nanocomposite, as shown in Fig. 18c, and d^107,108^.

This result is in a good agreement with various published reports on the interaction between MO NPs and pathogenic microorganisms by electrostatic potential, resulting in bacterial membrane detachment^107,109,110^.

A recent study reported that MO NPs could induce oxidative stress in pathogenic microbes^111^, and quickly-damage their cell membranes upon exposure to increased cellular ROS levels. In this study, the prepared nanocomposite was externally-linked to *E. coli* and *C. tropicalis* cells through electrostatic attraction and reduced the bacterial and yeast cell numbers via membrane leakages^109^. Our suggested action mechanism started with the adhesion of the nanocomposite to the exterior surface of *E. coli* and *C. tropicalis*. Then, Ti^2+^, Si^2+^ ions (from the external shell) and Fe^+2^ (from the core) penetrated the tested bacterial and yeast cells and destroyed their biological molecules, such as microbial mitochondria and DNA. After that, cellular toxicity due to oxidative tension and the generated ROS had been increased.

## Conclusion

Co_x_Ni_1−x_Fe_2_O_4_; x = 0.9/SiO_2_/TiO_2_ nanocomposite was prepared using a layer-by-layer approach. It was then decorated with C-dots synthesized using a one-pot hydrothermal method. The prepared nanocomposite was examined using several instruments to understand its phase, crystallinity, UV-absorption, band gap energy, surface area, pore size distribution, average size of particle, morphology and purity. The prepared nanocomposite was designed for wastewater treatment. Thus, two different applications were carried out: photocatalytic degradation of water pollutants, and disinfection of water-borne pathogens. Chloramine-T trihydrate was used as an example of organic pollutants in water, and many multi-drug-resistant bacteria and pathogenic fungi were employed as common water-borne microorganisms. Following this, the photocatalytic abilities of the prepared nanocomposite and the different factors (nanocomposite dose, chloramine-T initial concentration, and reaction pH) affecting their efficacy were studied. Our results showed that the photodegradation of chloramine-T followed second order kinetics. In addition, degradation mechanism suggested that holes had a significant role in the photodegradation via chloramine-T oxidation or forming free radicals. Moreover, the prepared nanocomposite showed more promising antimicrobial potential (high ZOI, low MIC) than bare C-dots, and our previously-reported nanocomposite (Co_x_Ni_1−x_Fe_2_O_4_; x = 0.9/SiO_2_/TiO_2_), suggesting a synergistic effect of C-dots with the nanocomposite. Notably, the antimicrobial ability of the prepared nanocomposite was significantly-increased after UV-irradiation. Above all, the synthesized nanocomposite showed a high ability for pathogenic-cell destruction as revealed by its good antibiofilm capabilities, suggesting a use for our nanocomposite in fighting multi-drug-resistant bacteria and fungi. Our work provides a revolutionary, nanomaterial-based and cost-effective solution for wastewater treatment to assist in solving global water shortage issues.

## Supplementary information

Supplementary Information.
