# Supplementary material for: Carbon-dot-loaded CoxNi1−xFe2O4; x = 0.9/SiO2/TiO2 nanocomposite with enhanced photocatalytic and antimicrobial potential: An engineered nanocomposite for wastewater treatment
Source: Sci Rep. 2020 Jul 13;10:11534. doi: 10.1038/s41598-020-68173-1 (PMC7358215; doi:10.1038/s41598-020-68173-1)
Supplement: Supplementary file 1 — Supplementary Information. [file 41598_2020_68173_MOESM1_ESM.docx]

**Carbon-dot-loaded Co_x_Ni_1-x_Fe_2_O_4_; x=0.9 / SiO_2_ / TiO_2_ nanocomposite with enhanced photocatalytic and antimicrobial potential: An engineered nanocomposite for wastewater treatment**

**M. Abd Elkodous^1, 2^†, Gharieb S. El-Sayyad^3, 4^†, Sally M. Youssry^1^, Hanady G. Nada^3^, Mohamed Gobara^4^, Mohamed A. Elsayed^4^, Ahmed M. El-Khawaga^4^, Go Kawamura^1, *^, Tan Wai Kian^5^, Ahmed I. El-Batal^3^ and Atsunori Matsuda^1, *^**

^1,^Department of Electrical and Electronic Information Engineering, Toyohashi University of Technology, 1-1 Hibarigaoka, Tempaku-cho, Toyohashi, Aichi 441-8580, Japan.

^2,^Center for Nanotechnology (CNT), School of Engineering and Applied Sciences, Nile University, Sheikh Zayed, Giza 16453, Egypt.

^3,^Drug Microbiology Lab, Drug Radiation Research Department, National Center for Radiation Research and Technology (NCRRT), Atomic Energy Authority, Cairo, Egypt.

^4,^ Chemical Engineering Department, Military Technical College (MTC), Egyptian Armed Forces, Cairo, Egypt

^5,^ Institute of Liberal Arts and Sciences, Toyohashi University of Technology, 1-1 Hibarigaoka, Tempaku-cho, Toyohashi, Aichi, 441-8580, Japan.

**† Equal Contribution**

***Corresponding Authors**

**1-Prof. Dr. Eng. Atsunori Matsuda**, Professor / Vice President (International Affairs) / Director of Center for International Education, Toyohashi University of Technology, 1-1 Hibarigaoka, Tempaku-cho, Toyohashi, Aichi 441-8580, Japan, Email: [matsuda.atsunori.hh@tut.jp](mailto:matsuda.atsunori.hh@tut.jp).

**2-Associate Prof**. Go Kawamura, Toyohashi University of Technology, Department of Electrical and Electronic Information Engineering, Toyohashi, Aichi 441-8580, Japan, Tel: +81-532-44-6796, Email: [kawamura.go.km@tut.jp](mailto:kawamura.go.km@tut.jp).


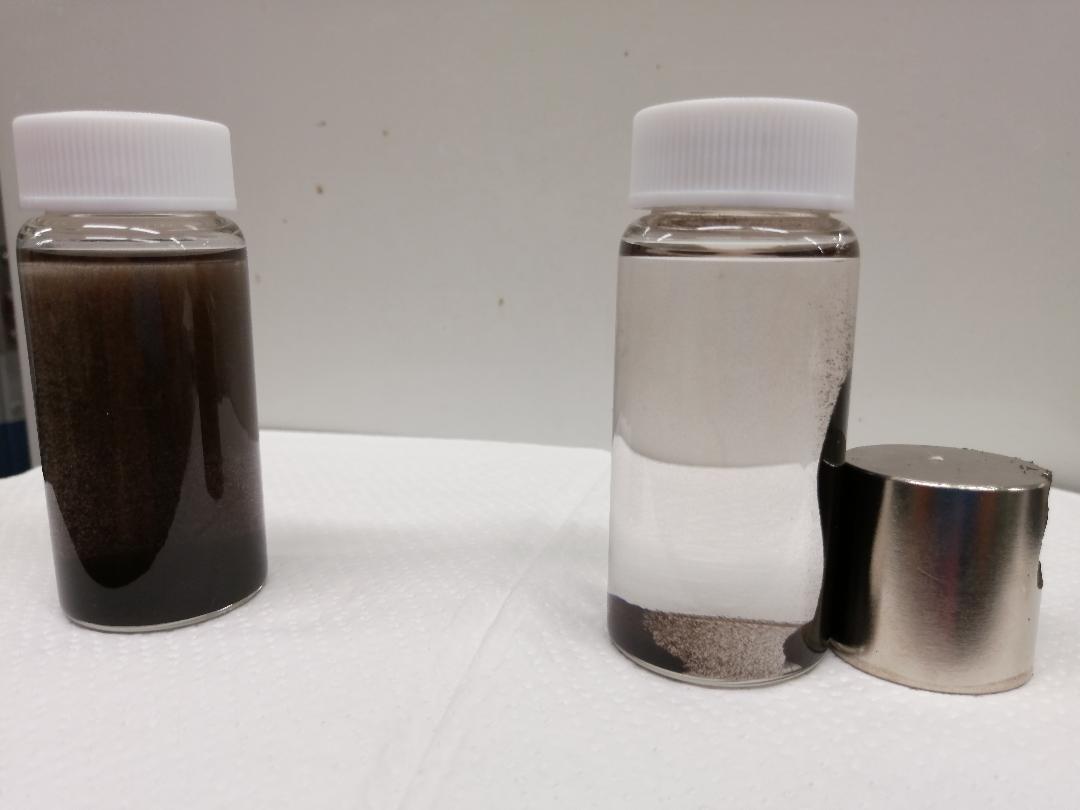

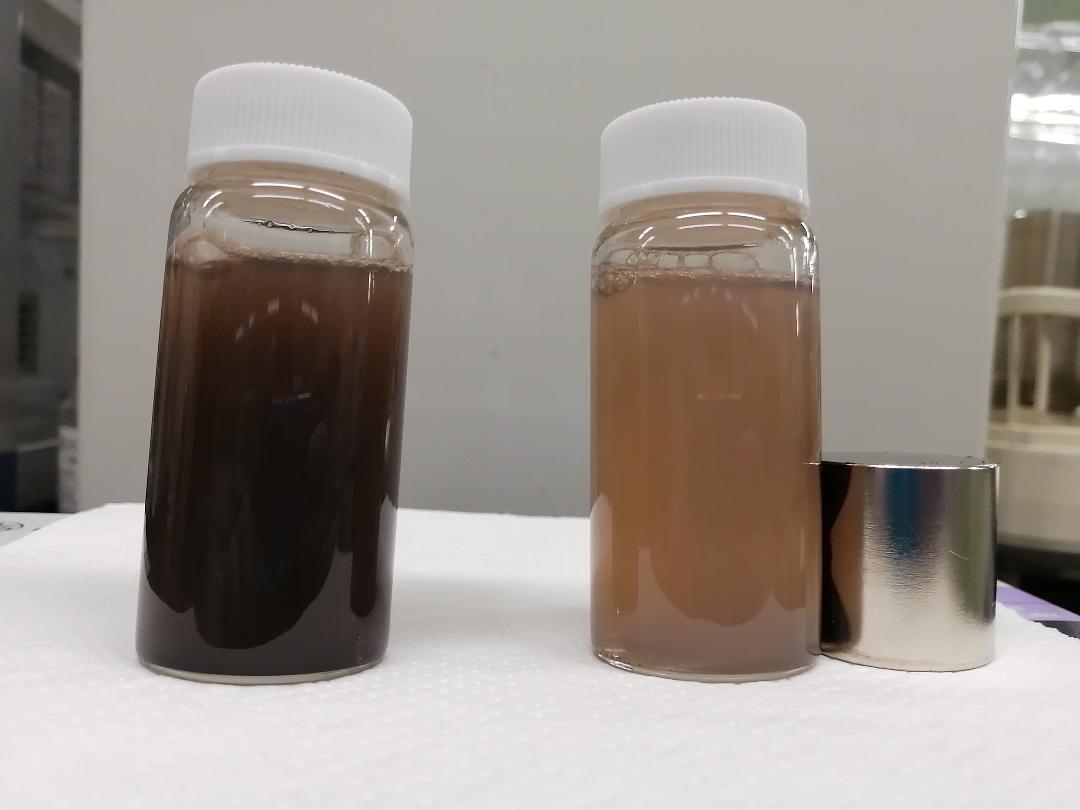


**(a)**

**(b)**

**Fig. S. 1:** Magnetic separation of a) Co_0.9_Ni_0.1_Fe_2_O_4_/SiO_2_ core shell structure and b) Co_0.9_Ni_0.1_Fe_2_O_4_/SiO_2_/TiO_2_ nanocomposite using 550 mT Nd magnet.

**
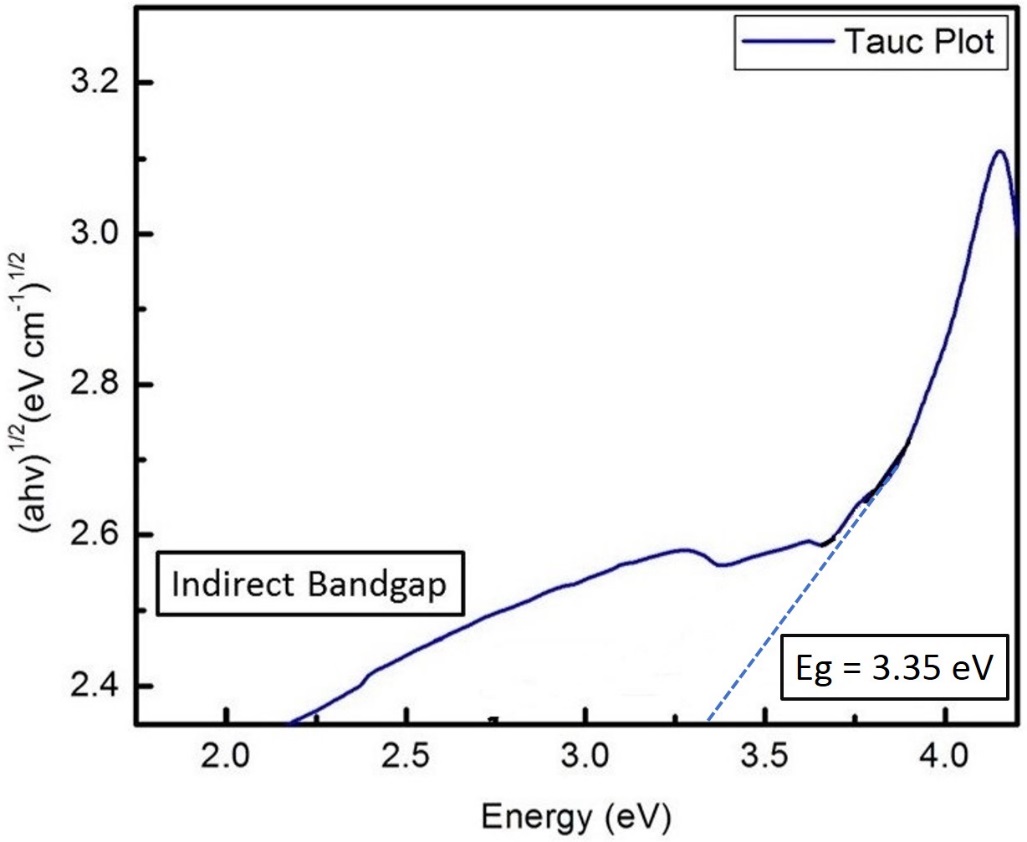
**

**Fig. S. 2:** Band gap calculation of the prepared nanocomposite using Tauc plot.
